# Evaluation of the Therapeutic Potential and Safety Profile of Six *Salvia* Species Native to Türkiye

**DOI:** 10.3390/plants15111718

**Published:** 2026-06-02

**Authors:** Nagehan Saltan, Fatmanur Tunç, Merve Baysal, Gamze Göger, Serkan Levent

**Affiliations:** 1 Department of Pharmaceutical Botany, Faculty of Pharmacy, Anadolu University, Eskişehir 26470, Türkiye; fatmanurt@anadolu.edu.tr; 2 Department of Pharmaceutical Toxicology, Faculty of Pharmacy, Anadolu University, Eskişehir 26470, Türkiye; mbaysal@anadolu.edu.tr; 3 Department of Pharmacognosy, Faculty of Pharmacy, Afyonkarahisar Health Sciences University, Afyonkarahisar 03030, Türkiye; gamze.goger@afsu.edu.tr; 4 Department of Analytical Chemistry, Faculty of Pharmacy, Anadolu University, Eskişehir 26470, Türkiye; serkanlevent@anadolu.edu.tr

**Keywords:** *Salvia*, antimicrobial activity, antioxidant activity, cytotoxic activity, LC-HRMS

## Abstract

The genus *Salvia* L. represents one of the most pharmacologically significant groups within the *Lamiaceae* family. This study investigates the phytochemical profiles and biological activities of six *Salvia* species native to Türkiye (*S. dorystaechas* B.T.Drew, *S. sclarea* L., *S. glutinosa* L., *S. tomentosa* Mill., *S. argentea* L., and *S. aethiopis* L.) to scientifically validate their extensive use in Turkish traditional medicine. Phytochemical characterization was performed using Liquid Chromatography–High-Resolution Mass Spectrometry (LC-HRMS), while biological potential was evaluated through antioxidant (DPPH), antimicrobial (MIC), and cytotoxicity (MTT on NIH/3T3 cells) assays. Among the taxa, *S. dorystaechas* exhibited the most potent antioxidant activity, with IC_50_ values of 0.090 mg/mL (infusion) and 0.072 mg/mL (ethanolic), which strongly correlated with high total phenolic contents (111.50 and 125.55 mg GAE/g, respectively). This species may also serve as a potential source of bioactive compounds. Antimicrobial screenings against pathogenic bacteria and *Candida* spp. demonstrated modest inhibitory effects, with MIC values ranging from 625 to >5000 µg/mL. Safety profiling indicated that the ethanolic extract of *S. tomentosa* showed the lowest cytotoxicity (IC_50_ 562.37 ± 49.50 µg/mL) among the tested samples, which nonetheless indicates a relatively narrow therapeutic window. LC-HRMS profiling revealed the presence of flavonoids and phenolic diterpenes, such as carnosol and rosmanol, providing a chemical rationale for the observed moderate activities. Consequently, rather than direct systemic pharmacological agents, these findings suggest that the studied *Salvia* species could serve as preliminary botanical sources for the isolation of specific secondary metabolites or for restricted topical applications.

## 1. Introduction

Globally, the *Lamiaceae* family represents a major botanical group, encompassing over 8000 species across 232 genera [1]. Türkiye stands as a critical genetic reservoir for this family, hosting 48 genera and 782 taxa, with a remarkable endemism rate of 44% [2]. Among these, the genus *Salvia* L. is particularly prominent, ranking second in Türkiye’s *Lamiaceae* flora with 107 taxa. Notably, approximately 10% of the world’s *Salvia* species are found in Türkiye, and more than half of these (54%) are endemic. This high level of biodiversity and endemism underscores Türkiye’s status as a vital evolutionary hub and offers unique opportunities for both taxonomic research and pharmacological exploration [1,2].

The *Lamiaceae* family includes plants used for a wide variety of purposes, including medicinal, food, spice, cosmetic, pharmaceutical, and ornamental purposes, from historical times to the present. In traditional medicine, these plants are frequently employed to treat gastritis, infections, dermatitis, bronchitis, and inflammatory disorders [3,4]. Ethnobotanical research in Türkiye demonstrates that *Lamiaceae* species are commonly used to address gastrointestinal, ear, nose, throat, and respiratory system disorders, primarily through infusion and decoction preparations [5]. According to Ertuğ et al., the most frequently used genera include *Sideritis* L., *Salvia, Thymus* L., *and Origanum* L., predominantly consumed as teas for conditions such as coughs, colds, and indigestion [6]. Additionally, another study identified the *Salvia* genus as the most represented among medicinally used *Lamiaceae* in Türkiye, with 37 taxa documented [5].

The therapeutic potential of major *Salvia* species, such as *S. officinalis* L., *S. sclarea* L., and *S. fruticosa* Mill., is internationally recognized and validated through their inclusion in various scientific monographs and pharmacopoeias, including the European and Turkish Pharmacopoeias [7,8,9,10,11,12].

Among natural resources, plants used for medicinal purposes exhibit various pharmacological effects due to the secondary metabolites they produce. In this context, the *Lamiaceae* family, which plays a significant role in essential oil production, contains numerous bioactive compounds, including terpenic compounds, flavonoids, lignans, coumarins, phenylpropanoids, iridoid glycosides, tannins, and fatty acids. The presence of these compounds confers antimicrobial, antiviral, antioxidant, anti-inflammatory, insect repellent, anthelmintic, hepatoprotective, antihypertensive, and analgesic properties on species belonging to this family [5]. *Salvia* species are primarily distributed across the Irano-Turanian and Mediterranean phytogeographic regions of Türkiye, exhibiting diverse morphological forms ranging from herbaceous to shrubby structures [13]. Beyond their botanical diversity, numerous *Salvia* taxa are deeply rooted in Turkish traditional medicine. According to a study [14], species such as *S. officinalis*, *S. sclarea*, *S. multicaulis* Vahl., *S. aethiopis*, and *S. tomentosa* are traditionally utilized for their sedative, antiseptic, and wound-healing properties, as well as for treating gastrointestinal disorders. Notably, *S. dorystaechas* holds significant ethnopharmacological value, with its leaves traditionally prepared as infusions to alleviate respiratory tract infections [15]. This therapeutic versatility is underpinned by a remarkably diverse chemical landscape; more than 150 polyphenolic compounds, including specialized phenolic acids and flavonoids, have been characterized within the genus [16]. The intricate synergy of these secondary metabolites provides the chemical rationale for the extensive biological activities and pharmacological potential associated with *Salvia* species [17].

The *Salvia* genus remains a cornerstone of pharmaceutical botany due to its diverse secondary metabolites and multi-target bioactivities. While foundational studies established the phenolic-antioxidant correlation in common species like *S. officinalis* [18], recent research has shifted towards unexplored endemic taxa and advanced analytical precision. Comparative characterizations have identified distinctive markers such as kaempferol in *S. brachyantha* (Bordz.) Pobed [19] and rutin in *S. transsylvanica* (Schur ex Griseb. & Schenk) Schur. [20]. Reflecting the latest trends, modern investigations now integrate high-resolution LC-MS metabolomics with chemometric tools such as PCA and hierarchical clustering to decode complex chemical scaffolds and validate the high antioxidant potential of species like *S. huberi* Hedge [21,22,23].

The selection of the *Salvia* species investigated in this study was guided by their extensive documentation in Turkish ethnomedicine. These taxa are widely utilized in traditional healing practices across Anatolia, primarily for treating respiratory infections, digestive disorders, and inflammatory conditions. To provide a comprehensive ethnobotanical context for our phytochemical and biological evaluations, the local names, utilized plant parts, preparation methods, and specific traditional medicinal indications of the studied species are summarized in Table 1.

Despite the extensive research on the *Salvia* genus, there remains a need for comprehensive comparative characterization of specific endemic taxa within the Anatolian flora. This study distinguishes itself by providing high-resolution metabolite profiling of six *Salvia* species (*S. dorystaechas*, *S. sclarea*, *S. glutinosa*, *S. tomentosa*, *S. argentea*, and *S. aethiopis*). Beyond traditional bioactivity screenings, the present research establishes a standardized multi-target evaluation by comparing traditional infusions and via LC-HRMS, including the under-investigated endemic *S. dorystaechas*. ethanolic extracts, integrated with a toxicological assessment on the NIH/3T3 cell line. By bridging high-resolution analytical techniques with safety–efficacy assessments under identical experimental conditions, this approach aims to eliminate the inter-laboratory variability found in fragmented literature. This approach is intended to facilitate the scientific validation for the ethnomedicinal applications of these species and offers new insights into their potential as natural pharmacological resources.

## 2. Results

### 2.1. Extract Yields

The extraction yields of the studied *Salvia* species, obtained through 5% infusion and 70% ethanol maceration, are summarized in Table 2. The yields for infusions (ranging from 10.67% to 14.71%) were consistently and significantly higher than those of the ethanolic extracts (2.97% to 3.62%).

Among the investigated taxa, *Salvia dorystaechas* provided the highest efficiency in both methods, yielding 14.71% (SD-I) and 3.62% (SD-E), respectively. The overall higher mass recovery in infusions can be attributed to the high polarity of water, which facilitates the extraction of bulk plant components, including primary metabolites such as mucilages and carbohydrates, alongside polar secondary metabolites. In contrast, the narrow and lower range observed in ethanolic extracts suggests a more selective recovery of specific secondary metabolites.

### 2.2. Total Phenolic Content and Antioxidant Activity

The antioxidant activity and total phenolic content (TPC) of the studied *Salvia* species are summarized in Table 2. According to the DPPH free radical scavenging assay, the ethanolic extracts (E) generally exhibited higher antioxidant potency, indicated by lower IC_50_ to their respective infusion (I) counterparts across all species, except *Salvia aethiopis* (SE).

Parallel to the antioxidant activity, TPC values were significantly higher in the ethanolic extracts. Among the tested taxa, *S. dorystaechas* extracts (SD-E and SD-I) emerged as the most potent, with SD-E yielding the highest concentration of phenolic compounds (125.55 ± 0.88 mg GAE/g) and the strongest radical scavenging activity (IC_50_: 0.07 ± 0.019 mg/mL). In contrast, the infusions of *S. argentea* (SA-I) and *S. sclarea* (SS-I) demonstrated the lowest TPC and antioxidant capacity among the samples. The extraction yields also varied notably, with infusions providing higher weight-to-weight recovery (10.67–14.71%) compared to ethanolic maceration (2.97–3.62%).

**Figure 1:**
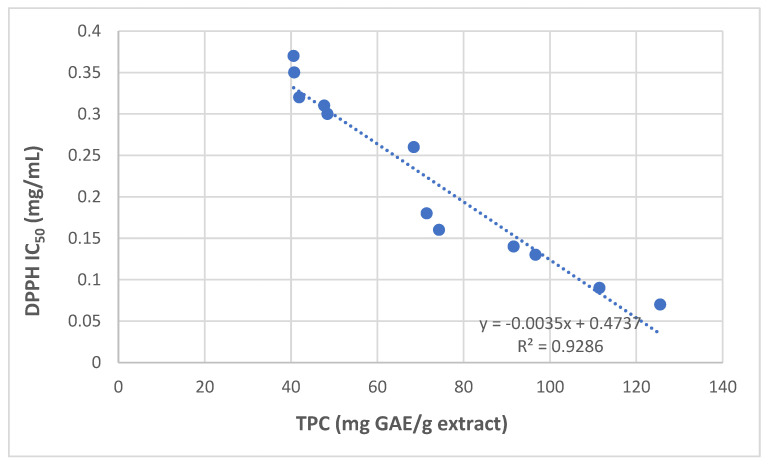
Regression analysis and scatter plot showing the correlation between total phenolic content (TPC) and DPPH radical scavenging activity (IC_50_) of the investigated *Salvia* extracts (r = −0.92, *p* < 0.001). The blue dots represent individual *Salvia* extracts, and the dashed line indicates the linear regression trendline (R^2^ = 0.9286).

Statistical analysis revealed significant differences (*p* < 0.05) among the studied *Salvia* species in terms of both phenolic content and antioxidant capacity. According to Tukey’s post hoc test, the ethanolic extract of *S. dorystaechas* (SD-E) exhibited the highest total phenolic content and the most potent antioxidant activity (IC_50_: 0.07 mg/mL), being statistically distinct from all other taxa (*p* < 0.05). However, a significant and strong negative correlation was observed between the total phenolic content and the DPPH radical scavenging activity (IC_50_) of the studied *Salvia* extracts (r = −0.92, *p* < 0.001). The linear relationship between these parameters was further quantified by a regression analysis, yielding the equation y = −0.0035x + 0.4737 (R^2^ = 0.9286) (Figure 1).

This strong correlation indicates a strong association between phenolic content and the observed antioxidant capacity. While these findings suggest that phenolic constituents contribute significantly to the redox-active profile of the investigated taxa, the overall bioactivity is likely influenced by a synergistic interplay with other identified secondary metabolites, such as phenolic diterpenoids (e.g., rosmanol and carnosic acid), which are also known for their potent radical scavenging properties.

### 2.3. Antimicrobial Activity

The antimicrobial activities of the *Salvia* species extracts were evaluated against a panel of pathogenic bacteria and fungi, including *Escherichia coli*, *Staphylococcus aureus*, *Candida tropicalis*, *C. albicans*, and *C. krusei*. The Minimum Inhibitory Concentration (MIC) values are presented in Table 3. Overall, the ethanolic extracts exhibited significantly stronger antimicrobial potency compared to the infusions against both bacterial and fungal strains. Among the infusions, *S. dorystaechas* (SD-I) demonstrated moderate antibacterial activity against *E. coli* and *S. aureus* (MIC: 1250 µg/mL) and the most notable antifungal effect against *C. tropicalis* (MIC: 625 µg/mL). In contrast, most other infusion extracts displayed limited or no inhibitory effects, with MIC values typically ≥2500 µg/mL for bacteria and ≥5000 µg/mL for *Candida* species.

The ethanolic extracts demonstrated markedly lower MIC values, indicating higher potency. *S. sclarea* (SS-E) exhibited the most robust antibacterial effect against *E. coli* (MIC: 625 µg/mL). Regarding antifungal activity, SG-E, SD-E, SA-E, and ST-E showed substantial inhibitory effects against *C. tropicalis* with MIC values ranging between 625 and 1250 µg/mL. Notably, SD-E and SG-E also inhibited the growth of *C. albicans* and *C. krusei* at relatively low concentrations (MIC: 625–1250 µg/mL), whereas their respective infusions were largely inactive. As expected, the reference agents Ciprofloxacin and Fluconazole maintained significantly lower MIC values, validating the experimental setup.

The results suggest that the antimicrobial constituents of *Salvia* species, likely including non-polar terpenoids and specific phenolics, are more efficiently recovered through ethanolic extraction. These findings highlight the potential of organic *Salvia* extracts as sources of natural antimicrobial agents.

### 2.4. Cytotoxicity Activity

As a well-characterized and non-tumorigenic cell line, NIH/3T3 is frequently utilized for high-throughput in vitro toxicity screening of experimental therapeutics [39,40]. This cell line provides a robust platform for preliminary safety evaluations, particularly when investigating natural product-based therapeutic candidates.

Activity evaluations in antimicrobial activity studies in the literature are generally based on MIC values, although it is important to note that classification thresholds can vary significantly depending on the study’s scope and the standards adopted. While MICs for conventional antibiotics typically fall within the range of 0.01 to 10 µg/mL, plant extracts are generally considered to have antimicrobial potential when their MIC values range between 100 and 1000 µg/mL [41]. In the context of traditional medicine, plant extracts with MIC values below 8 mg/mL are generally regarded as biologically active. To provide a structured evaluation in this study, we adopted the criteria where extracts exhibiting MIC values below 100 µg/mL are considered to possess strong antibacterial activity, while those with MICs between 100 and 625 µg/mL show moderate activity, and values exceeding 625 µg/mL are indicative of weak or low antibacterial potential [42].

Similarly, the categorization of antioxidant potency via DPPH scavenging activity is subject to interpretation across different research contexts. In this study, IC_50_ values are categorized based on widely cited literature as follows: very strong activity for values below 10 µg/mL, strong activity for values between 10 and 50 µg/mL, moderate activity for values between 50 and 100 µg/mL, weak activity for values between 100 and 250 µg/mL, and no activity for values above 250 µg/mL [43]. These categorized thresholds serve as a benchmark for comparing our findings with existing ethnobotanical and phytochemical data.

To evaluate the cytotoxic effects and preliminary selectivity of the promising samples, four extracts (SD-I, SD-E, ST-I, and ST-E) were selected for cytotoxicity screening on the NIH/3T3 non-tumorigenic cell line, based on their comparatively higher antioxidant capacities together with relatively more notable antimicrobial activity among the tested extracts. The IC_50_ values determined after 24 h of exposure, together with the calculated Selectivity Index (SI) values, are summarized in Table 4. Since multiple MIC values were obtained for each extract against different microorganisms, both SI_min_ and SI_max_ values were calculated. Among the tested plant extracts, ST-E (*S. tomentosa* ethanol extract) exhibited the highest IC_50_ value (562.37 ± 49.50 µg/mL) and the highest SI values (SI_min_: 0.45; SI_max_: 0.90), indicating lower cytotoxicity relative to the other tested extracts and comparatively better selectivity. In contrast, SD-E (*S. dorystaechas* ethanol extract), despite showing the strongest antioxidant activity in previous assays, demonstrated the highest cytotoxicity with an IC_50_ of 110.06 ±0.31 µg/mL and lower SI values (SI_min_: 0.02; SI_max_: 0.18). The infusion extracts SD-I and ST-I showed moderate cytotoxic effects, with IC_50_ values of 173.82 ±25.09 µg/mL and 408.02 ±4.63 µg/mL, respectively. Overall, the calculated SI values remained below 1, suggesting limited selectivity of the tested extracts toward microbial cells relative to mammalian cells under the evaluated experimental conditions [44].

### 2.5. Phytochemical Profiling

The LC-HRMS analysis of 70% ethanol (E) and infusion (I) extracts from six *Salvia* species identified nine key phytochemical compounds (Table 5) (Supplementary Figures S1–S15). Luteolin, luteolin-7-O-glucuronide, and apigenin were the most prevalent constituents, detected in both the ethanol and infusion extracts of *S. argentea* (SA), *S. tomentosa* (ST), and *S. sclarea* (SS). In contrast, these compounds were only identified in the infusion extract of *S. aethiopis* (SE). In *S. dorystaechas* (SD), luteolin-7-O-glucuronide was the only flavonoid present in both extraction types.

*S. tomentosa* (ST) exhibited the highest chemical diversity among the studied species, with cynaroside (luteolin-7-O-glucoside) being uniquely detected in this taxon. *S. dorystaechas* (SD-E) was distinguished as the only species containing the polyphenol rosmanol, while rosmarinic acid was exclusively detected in *S. glutinosa* (SG). Regarding terpenoids, carnosol was identified in all species except *S. aethiopis*. Overall, the phytochemical profiling indicated that ethanol extracts were richer in terpenoids and phenolic compounds compared to the infusions.

## 3. Discussion

The phytochemical diversity and biological orientation of the *Salvia* genus are heavily influenced by the extraction solvent’s polarity and the specific genetic makeup of the taxa. In this study, 70% ethanol generally outperformed infusions in recovering both phenolic compounds and terpenoids, a phenomenon attributed to the intermediate polarity of the hydroethanolic mixture, which facilitates the dissolution of a broader range of secondary metabolites, including methylated flavonoids and diterpenoids, such as carnosol.

A direct correlation was observed between Total Phenolic Content (TPC) and DPPH radical scavenging activity, reinforcing the role of polyphenols as primary redox-active constituents in *Salvia*. The most potent antioxidant source was *S. dorystaechas* (SD), whose ethanolic extract had TPC values greater than 125 mg GAE/g. Based on the categorization by [43] the DPPH IC_50_ values observed in our study, particularly for *S. dorystaechas,* fall within the ‘strong’ to ‘moderate’ activity ranges. This high activity is likely linked to the presence of rosmanol and carnosic acid, which were uniquely identified in this species. Compared to conventional antioxidants, rosmanol, a phenolic diterpene, is renowned for its exceptional capacity to donate hydrogen. Furthermore, in our study, 70% ethanol was more selective for recovering antioxidant-rich fractions, aligning with the report [45].

Our findings for *S. sclarea* and *S. aethiopis* are in partial agreement with the literature [46]; indeed, dynamic interspecific variation and modern technological platforms heavily shape the bioactivity profiles of these specific taxa. For instance, recent studies utilizing *S. aethiopis* and *S. sclarea* extracts for the green synthesis of silver nanoparticles (AgNPs) demonstrate that advanced formulation significantly enhances baseline antioxidant and anti-inflammatory pathways governed by phenolics like isoquercetin and naringenin [47]. However, the superior TPC values in our Turkish *S. dorystaechas* samples highlight the high-yield bioactive potential of Türkiye’s flora. These discrepancies between our results and those of the study [37] for *S. glutinosa* further underscore the profound impact of locality-dependent variations and genetic factors on the genus’s chemical profile. Geographic and regional variations contribute to distinct chemical profiles even within the same species; while our LC-HRMS profiling focused on other markers, recent investigations on *S. glutinosa* from the Artvin region revealed coumaric acid as a predominant compound, highlighting strong solvent-dependent antimicrobial potencies against strains like *S. aureus* that surpass commercial standards [48]. The high reducing potential of the extracts is strongly correlated with their polyphenolic architecture, as higher TPC values typically facilitate greater antioxidant activity [49]. This correlation was evident in our results, where the taxa with the highest phenolic content consistently demonstrated the lowest IC_50_ values in DPPH assays. In the present study, although common phenolic acids frequently reported in the *Salvia* genus, including caffeic, ferulic, and *p*-coumaric acids, were targeted during LC-HRMS screening, they were not detected in the investigated extracts, possibly due to their occurrence at trace levels below the detection limits under the applied experimental conditions. Instead, rosmarinic acid, together with a diverse range of flavonoids (Table 5), represented the predominant polyphenolic constituents of the analyzed species.

Interestingly, rosmarinic acid was only found in *S. glutinosa* in our investigation, despite the fact that it is frequently mentioned as a primary biomarker for the *Salvia* genus. This implies that rather than rosmarinic acid alone, the antioxidant capacity in other species, such as *S. tomentosa* and *S. argentea*, is likely driven by a synergistic mix of flavonoids (luteolin, apigenin) and diterpenes. This multi-component synergy and phytochemical fluctuation are well-documented across the genus. Ethnobotanical surveys and in vivo trials on *S. argentea* validate its traditional therapeutic window, proving that its rosmarinic acid- and salvigenin-rich matrices exert robust anti-inflammatory responses by downregulating NF-κB and cytokine pathways without acute oral toxicity [26,50]. Concurrently, recent work on *Salvia tomentosa* Miller reinforces that this secondary metabolite density is highly organ-dependent and subject to cultivation factors, where combinations of aerial parts (stems, leaves, flowers) or wild versus cultivated matrices distinctly alter baseline free radical scavenging and antimicrobial outputs [51,52]. The lower IC_50_ values of ethanol extracts (ranging from 0.07 to 0.32 mg/mL) compared to infusions highlight the necessity of organic solvents for maximizing the recovery of these bioactive fractions.

The antimicrobial results revealed that *Salvia* extracts are particularly effective against fungal pathogens, specifically *Candida tropicalis*. The biological potential of these extracts can be evaluated based on established potency thresholds; according to the report [42], plant extracts with MIC values between 100 and 625 µg/mL are considered to show moderate antibacterial activity. However, although the extracts demonstrated measurable antimicrobial activity, the obtained MIC values in this study generally indicated weak to moderate activity according to commonly accepted criteria for crude plant extracts. In particular, MIC values above 1000 µg/mL may limit the direct practical applicability of the crude extracts as potent antimicrobial agents. The antimicrobial activity observed in the present study should therefore be interpreted cautiously, especially considering that crude plant extracts often contain complex mixtures of active and inactive constituents that may reduce the apparent biological potency. Similar MIC ranges have been reported in previous studies investigating crude botanical extracts, where moderate antimicrobial effects were frequently associated with the presence of phenolic compounds, flavonoids, terpenoids, and other secondary metabolites. The ethanolic extracts of *S. sclarea* (SS-E) and *S. glutinosa* (SG-E) exhibited notable inhibitory effects, which can be attributed to the presence of carnosol and acacetin. Although the specific in vitro or molecular mechanisms of action were not directly investigated in the present study, the antimicrobial activity of the *Salvia* extracts can be theoretically attributed to the well-documented mechanisms of their major secondary metabolites, such as luteolin, rosmarinic acid, and carnosol, identified via LC-HRMS. Luteolin, a prominent flavonoid, has been reported to intercalate between the polar head groups and hydrophobic regions of bacterial cytoplasmic membranes, altering membrane fluidity and thickness, which ultimately leads to the disruption of membrane integrity and cell death [53]. Similarly, rosmarinic acid is known to exert significant antimicrobial and antibiofilm effects by inducing structural alterations in the microbial cell membrane, causing leakage of intracellular contents and reducing mitochondrial activity in fungal strains [54]. Furthermore, diterpenes like carnosol and carnosic acid have been shown to act as efflux pump modulators by dissipating the membrane potential gradient, thereby compromising bacterial survival and potentiating antimicrobial susceptibility [55]. Therefore, the limited to moderate MIC values observed in our study likely result from a synergistic or additive structural disruption of microbial membranes driven by these phytochemicals. These extracts should not be considered as primary antibiotic or antifungal agents, but rather as potential supportive phytotherapeutic agents, especially in topical applications, or as sources of secondary metabolites for further purification. However, the relatively high MIC values observed in this study indicate that additional purification and fractionation are needed to identify the specific compounds responsible for the antimicrobial activity. This relatively moderate efficacy may be attributed to the nature of the crude extracts, where the absolute concentration of potent antimicrobial markers, such as carnosic acid or carnosol, may be diluted by the presence of other non-active matrix components. Furthermore, the 70% ethanol used in this study, while excellent for recovering a broad range of polyphenols, might not have fully optimized the recovery of highly lipophilic constituents that often possess stronger antimicrobial properties. Future studies utilizing alternative extraction methods, such as supercritical fluid extraction (SFE) or microwave-assisted extraction, could potentially enhance the yield of these specific antimicrobial fractions and provide a clearer understanding of the genus’s maximal inhibitory potential.

The therapeutic index (selectivity) is a crucial component of pharmacological evaluation. As a non-tumorigenic cell line, NIH/3T3 provides a robust platform for preliminary safety evaluations of natural product-based candidates [39,40]. In our study, a critical balance between efficacy and toxicity was observed. *S. dorystaechas* (SD-E) exhibited substantial cytotoxicity in NIH/3T3 cells (IC_50_: 110.06 µg/mL), although demonstrating the highest antioxidant and antimicrobial activities. These differences in cytotoxicity (Table 4) appear to be related to the specific phytochemical compositions identified (Table 5). The lower IC_50_ value of SD-E (110.06 µg/mL) could be associated with its diterpenoid profile, notably the presence of rosmanol, carnosol, and carnosic acid. In the literature, these phenolic diterpenoids are reported to exhibit dose-dependent effects, where higher concentrations may lead to cellular stress in non-tumorigenic lines like NIH/3T3. This suggests that its bioactive compounds may lack specificity at higher concentrations.

In contrast, *S. tomentosa* (ST-E) emerged as a promising candidate showing a higher IC_50_ value (562.37 µg/mL), which might be due to a different balance between its flavonoid and diterpenoid constituents, such as the presence of cynaroside. These findings suggest that specific chemical markers, rather than total phenolic content alone, play a role in the safety profile of these extracts. This favorable selectivity and balanced safety profile of *S. tomentosa* are supported by recent literature; for instance, metabolic profiles of *S. tomentosa* organs (roots, aerial parts, and inflorescences) predominated by rosmarinic and salvianolic acids demonstrated minimal cytotoxicity toward reference murine fibroblasts while simultaneously exerting potent, targeted anti-proliferative effects against human AGS gastric adenocarcinoma epithelial cells [56]. While extracts with MIC values below 1000 µg/mL are generally considered to have significant antimicrobial potential [41], the high cytotoxicity observed in some potent fractions can limit their application. Despite its moderate bioactivity, the high IC_50_ value of ST-E on NIH/3T3 cells (562.37 ± 49.50 µg/mL) indicates a lower toxic potential compared to SD-E, aligning with the requirement for non-toxic natural therapeutics. The phytochemical profiling of this species, characterized by the presence of cynaroside (luteolin-7-O-glucoside), likely contributes to its balanced bioactivity–toxicity ratio.

Furthermore, the biological activities observed in this study provide a potential scientific basis for the traditional medicinal applications of these taxa, as summarized in Table 1. The notable antioxidant and antimicrobial potencies, particularly against fungal pathogens, align with the ethnomedicinal use of *S. dorystaechas*, *S. tomentosa*, and *S. sclarea* in treating diseases potentially related to microbial infections. The prevalence of specific secondary metabolites, such as carnosol and flavonoids identified via LC-HRMS, likely contributes to the therapeutic efficacy reported in Anatolian folk medicine. By exploring these centuries-old practices through modern high-resolution profiling, our findings reinforce the importance of documenting and preserving traditional knowledge as a roadmap for discovering new natural pharmacological agents. The high correlation coefficient (r = −0.92) found in our study aligns with previous reports on the *Salvia* genus, a strong association between the phenolic density and the free radical scavenging capacity. This relationship highlights the significant role of phenolic constituents in the potent antioxidant activity observed in *S. dorystaechas*. These results support the view that the rich phenolic and terpenoid profile of these species contributes to their therapeutic potential in treating oxidative-stress-related disorders.

The pharmacological potential of the *Salvia* genus is largely attributed to its diverse secondary metabolite profile, primarily categorized into phenolic compounds and terpenoids. Beyond their aromatic properties, these species serve as complex natural reservoirs for potent phenolics like rosmarinic acid and various diterpenoids. Current scientific literature supports traditional uses of *Salvia*, demonstrating that inter-species variations lead to distinct chemical fingerprints [57,58]. This extensive chemical diversity is consistent with the evolutionary success of the *Salvia* genus, which comprises over 1024 species globally distributed across major biodiversity hotspots. Comprehensive reviews on *S. sclarea* highlight its vast ethnobotanical legacy in treating digestive, menstrual, and inflammatory disorders, linking these traditional applications to an intricate reservoir of secondary metabolites, particularly oxygenated monoterpenoids like linalyl acetate and linalool, alongside diverse flavonoids and phenolic acids [59]. Our results indicate that this phytochemical composition is dynamic and influenced by several factors, including genetic phylogeny, geographic origin, the extraction methodology, and localized ecological conditions. The prevalence of specific markers, such as the unique presence of rosmanol in *S. dorystaechas* or cynaroside in *S. tomentosa*, ultimately dictates the efficacy and variability observed in the biological activity studies [60,61]. Furthermore, the higher recovery of terpenoids in ethanol extracts confirms that the choice of solvent significantly impacts the plant.

The variations observed in TPC and MIC values compared to previous reports [37,46] underscore the impact of Anatolian geography on *Salvia* phytochemistry. Localized ecological pressures often lead to the enhanced biosynthesis of specific secondary metabolites as defensive mechanisms, resulting in the chemical fingerprints observed in our LC-HRMS analysis. The detection of acacetin and carnosic acid primarily in ethanol extracts further supports the conclusion that the traditional use of infusions may not fully capture the genus’s entire pharmacological potential, particularly regarding non-polar terpenoids. While this study provides a high-resolution phytochemical and biological characterization of these six *Salvia* species, certain limitations remain. First, the biological activities were evaluated using crude extracts; hence, the potential synergistic or antagonistic interactions between individual compounds have yet to be fully elucidated through bio-guided isolation. Second, the current safety evaluations are limited to *in vitro* NIH/3T3 cell lines, necessitating future *in vivo* studies to confirm the systemic safety, pharmacokinetics, and bioavailability of these taxa.

Future research directions should involve the isolation of specific biomarkers identified in our LC-HRMS analysis, such as rosmanol, acacetin, and cynaroside, to investigate their molecular mechanisms of action. Additionally, further investigations including minimum bactericidal concentration (MBC), minimum fungicidal concentration (MFC), and transcriptomic studies would be necessary to better characterize their exact mechanistic pathways and mode of action.

## 4. Materials and Methods

### 4.1. Plant Material and Extraction

The plant specimens investigated in this study were collected from their natural habitats in Türkiye during the 2024 flowering season. The specimens were identified by the authors and prepared as herbarium materials, and are preserved in the Anadolu University Faculty of Pharmacy Herbarium (ESSE). The studied taxa and their respective accession numbers are as follows: *S. dorystaechas* B.T.Drew (ESSE 16433), *S. sclarea* L. (ESSE 16347), *S. glutinosa* L. (ESSE 16311), *S. tomentosa* Mill. (ESSE 16348), *S. argentea* L. (ESSE 16346), and *S. aethiopis* L. (ESSE 16432). Extracts were prepared from the flowering aerial parts of the plants using two different methods—5% infusion (aqueous) and 70% ethanol extraction—following the protocol described in the literature [62].

The extraction efficiency for each species was determined gravimetrically and expressed as a percentage yield (*w/w*) relative to the initial dried plant material. The yield was calculated using the following equation:

$$\mathrm{Yield}\;\left(\%\right)=\left(A÷A1\right)\times 100$$

A denotes the final mass of the dried extract obtained after solvent evaporation. A1 represents the initial mass of the pulverized plant material used in the extraction process.

### 4.2. Assessment of Total Phenolic Content and Antioxidant

#### 4.2.1. Determination of Total Phenolic Content (TPC)

The total phenolic content of the extracts was quantified using the Folin–Ciocalteu reagent (FCR) (Merck, Darmstadt, Germany) as described in the literature [63]. To establish a standard for quantification, a linear calibration curve was constructed using gallic acid working solutions within a concentration range of 0.03 to 1 mg/mL. The absorbance of each standard and sample was recorded at 760 nm using a BioTek microplate spectrophotometer (Winooski, VT, USA). The resulting regression equation was utilized to determine the phenolic concentrations of the *Salvia* extracts. All results are expressed as milligrams of gallic acid equivalents per gram of extract (mg GAE/g).

#### 4.2.2. DPPH Radical Scavenging Activity

The antioxidant capacity of the aerial part extracts was assessed using the 2,2-diphenyl-1-picrylhydrazyl (DPPH) radical scavenging assay, following the methodology detailed in [64]. The radical scavenging effect (Inhibition %) was calculated using the following equation:

$$\mathrm{Inhibition}\;\left(\%\right)=\left[\left(\mathrm{Acontrol}-\mathrm{Asample}\right)÷\mathrm{Acontrol}\right]\times 100$$

Acontrol is the absorbance of the control (containing all reagents except the sample), and Asample is the absorbance of the extract. The IC_50_ values (the concentration required to inhibit 50% of DPPH radicals) were calculated through nonlinear regression analysis of the dose–response data. All extract concentrations used for IC_50_ calculations were precisely determined based on the dry weight of the extract (mg dry extract/mL). Curve fitting and statistical modeling were performed using SigmaPlot 13.0 (Systat Software Inc., San Jose, CA, USA). To ensure reproducibility, all assays were conducted in triplicate, and the results are expressed as the mean values of independent experimental runs.

### 4.3. Assessment of Antimicrobial Activity

#### 4.3.1. Microbial Strains and Media

The antimicrobial potential of the *Salvia* extracts was evaluated against a panel of microorganisms, including *Staphylococcus aureus* ATCC 6538, *Escherichia coli* ATCC 8739, and the yeast strains *Candida albicans*, *C. tropicalis*, and *C. krusei*. Bacterial strains were cultured in Mueller–Hinton Broth (MHB, Sigma-Aldrich, Biolife Italiana, Milan, Italy), while yeasts were maintained in RPMI-1640 medium with L-glutamine, buffered to pH 7.0 with 3-[N-morpholino]-propanesulfonic acid (MOPS) (Sigma-Aldrich, St. Louis, MO, USA). Ciprofloxacin (Sigma-Aldrich, St. Louis, MO, USA) and fluconazole (Sanovel Pharmaceutical Industry, Istanbul, Türkiye) were employed as standard reference drugs.

#### 4.3.2. Broth Microdilution Method

The Minimum Inhibitory Concentrations (MICs) were determined using the broth microdilution method, conducted in accordance with Clinical and Laboratory Standards Institute (CLSI) guidelines M7-A7 for bacteria [65] and M27-A2 for yeasts [66] with minor modifications. Extracts were tested within a concentration range of 78.125 to 5000 µg/mL, while reference drugs were prepared at concentrations ranging from 0.5 to 64 µg/mL.

Standardized inocula were prepared from overnight cultures to yield final concentrations of 10^6^ colony-forming units (CFU/mL) for bacteria and 1–2 × 10^3^ cells/mL for yeasts. The assays were performed in 96-well microplates. Following inoculation, the microplates were incubated for 24 h at 37 °C for bacteria and 48 h at 28 °C for yeasts. Positive growth controls (wells containing medium and inoculum without extracts) were included to ensure microbial viability.

To facilitate the visual detection of microbial growth, 20 μL of a 0.01% resazurin solution was added to each well. The MIC was defined as the lowest concentration of the extract that prevented a color change (from blue to pink) or visible turbidity, indicating the inhibition of metabolic activity. All experiments were performed in triplicate to ensure the reproducibility of the results.

### 4.4. Assessment of Cytotoxicity by MTT Assay

#### 4.4.1. Cell Culture Conditions

The NIH/3T3 mouse embryonic fibroblast cell line (ATCC CRL-1658™) was utilized to evaluate the safety profile of the selected extracts. Cells were maintained in a humidified incubator at 37 °C with 5% CO_2_. The growth medium consisted of high-glucose DMEM (89%), supplemented with 10% fetal bovine serum (FBS) and 1% penicillin–streptomycin. All assays were conducted using cells between passages 2 and 6 to ensure phenotypic stability.

#### 4.4.2. MTT Viability Assay

The cytotoxic effects of four extracts, *S. dorystaechas* infusion (SD-I), *S. dorystaechas* ethanol (SD-E), *S. tomentosa* infusion (ST-I), and *S. tomentosa* ethanol (ST-E), based on their comparatively higher antioxidant activities, together with relatively more notable antimicrobial activity among the tested samples, and to enable comparison between infusion and ethanol extracts of the same species, were investigated in NIH/3T3 healthy cells. Cell viability was assessed using the 3-(4,5-dimethylthiazol-2-yl)-2,5-diphenyltetrazolium bromide (MTT) colorimetric assay, which correlates mitochondrial metabolic activity with the formation of insoluble formazan crystals [67].

The MTT assay was performed in 96-well microplates, with each concentration tested in quadruplicate, and all experiments were independently repeated three times. Stock solutions of the extracts were freshly prepared in dimethyl sulfoxide (DMSO) at the highest concentrations at which they were fully soluble, then diluted with culture medium and sterilized using a 0.22 µm filter, and further diluted with culture medium to achieve test concentrations (39.06 to 625 µg/mL). A 1% DMSO solution was used as the vehicle control. Cells were detached using trypsin–EDTA and centrifuged, and the resulting pellet was resuspended in complete medium. The viable cell count was determined by trypan blue exclusion. Cells were seeded at a density of 1 × 10^4^ cells/100 µL per well into 96-well plates and incubated for 24 h to allow attachment. Following incubation, the test compounds were added at their respective concentrations and incubated for an additional 24 h. After the treatment period, the media were aspirated, and cells were incubated with 0.5 mg/mL MTT solution for 3 h. The resulting formazan crystals were solubilized in 100 µL of DMSO, and absorbance was measured at 540 nm using a microplate reader. Cell viability was calculated using the formula:

% Cell viability = [(OD_Substance_ − OD_Control_)/OD_Control_] × 100.

OD: Optical Density.

The percent viability was determined for each concentration, and the half-maximal inhibitory concentration (IC_50_) values were calculated using linear or non-linear curve fitting methods [68].

Selectivity Index (SI) values were calculated as the ratio of IC_50_ values obtained in NIH/3T3 cells to MIC values determined in antimicrobial assays. SI_min_ and SI_max_ were calculated using the highest and lowest MIC values obtained for each extract, respectively [44].

### 4.5. Phytochemical Profiling via LC-HRMS Analysis

Phytochemical characterization of the *Salvia* extracts was performed using a high-resolution Shimadzu LC-MS-IT-TOF (Kyoto, Japan) system. The liquid chromatography (LC) module consisted of two LC-20AD binary pumps, a DGU-20A3R degasser, a CTO-10ASvp column oven, a SIL-20AC autosampler, and an SPD-M20A photodiode array (PDA) detector. Chromatographic separation was achieved on an Inertsil ODS-3 column (150 mm × 1.5 mm, 5 µm, C-18 packing). The mobile phase comprised acetonitrile and water with 0.1% formic acid, delivered at 0.15 mL/min. The column oven temperature for the samples sent in 3 µL injection volumes was optimized as 40 °C, CDL temperature as 200 °C, heat block temperature as 200 °C, and nebulizer gas flow as 1.5 L/min. The acquired high-resolution m/z values were evaluated by comparison with literature data and reference databases, enabling the tentative identification of phytochemical constituents.

### 4.6. Statistical Analysis

All experiments were conducted using three independent biological replicates, each performed in triplicate to ensure reproducibility. Results are expressed as mean ± standard deviation (sd). Before performing One-way Analysis of Variance (ANOVA), the data were evaluated for normality using the Shapiro–Wilk test and for homogeneity of variance using Levene’s test. Statistical differences between the *Salvia* species and their extracts were determined via ANOVA followed by Tukey’s post hoc test for multiple comparisons. The IC_50_ values for antioxidant activities were calculated individually for each independent experiment using non-linear regression analysis, and the final values represent the mean of these independent runs. Pearson’s correlation coefficient (r) was calculated to evaluate the relationship between total phenolic content (TPC) and IC_50_ values across all studied extracts. Statistical significance was set at *p* < 0.05. All statistical analyses were conducted using IBM SPSS Statistics for Windows, Version 24.0 (IBM Corp., Armonk, NY, USA).

## 5. Conclusions

This study provided a comprehensive evaluation of infusions and 70% ethanol extracts from six *Salvia* species with established ethnomedicinal backgrounds. Our findings demonstrate a significant correlation between antioxidant potency and total phenolic content across the studied taxa. Phytochemical characterization through LC-HRMS confirmed that the observed biological effects are associated with specific phenolic constituents and terpenoids.

Regarding antimicrobial efficacy, the results of the present study demonstrated that the investigated extracts possess weak to moderate activity against the tested microorganisms. Although the observed activity does not indicate strong antimicrobial potency at the crude extract level, the findings suggest that the extracts may contain bioactive constituents worthy of further investigation. In this context, the calculated selectivity index (SI) values provided preliminary information regarding the balance between antimicrobial activity and cytotoxicity, contributing to a more comprehensive biological evaluation of the extracts.

Among the evaluated samples, the ST-E (*S. tomentosa* ethanol extract) exhibited a balanced profile, combining notable bioactivity with comparatively lower cytotoxicity in NIH/3T3 cells. These results highlight the influence of solvent selection on the recovery of bioactive secondary metabolites and provide a scientific basis for the potential development of *S. tomentosa* as a natural therapeutic agent. However, future studies involving bioactivity-guided fractionation, isolation of active compounds, MBC/MFC determinations, and mechanistic analyses are required to better clarify the antimicrobial potential and pharmacological relevance of these extracts.

In conclusion, our findings contribute to the transition of fragmented ethnomedicinal knowledge toward a more validated pharmacological framework. By evaluating phytochemical profiles alongside biological activities and preliminary toxicological safety, this study offers a standardized approach for the evidence-based use of *Salvia* species. This comparative framework provides a reliable basis for future research into drug discovery and the sustainable utilization of Turkey’s endemic plant biodiversity.

## Figures and Tables

**Table 1:** Ethnobotanical information: local names and traditional medicinal uses of the studied *Salvia* species.

Species	Local Name	Part Used/Preparation	Traditional Uses	References
*S. sclarea* L.	Misk adaçayı, Ayıkulağı, Tüylü adaçayı, Paskulak	Aerial parts, leaves, flowers, powder, infusion, essential oil	Alleviation of symptoms in cold, cough, influenza, bronchitis, and asthma; sedative effects for gastric spasms, constipation.	[4,13,24,25]
*S. argentea* L.	Gümüş şalba, Boz şalba	Aerial parts, leaves, rhizomes, whole plant, powder, decoction, infusion	Respiratory, digestive, and hemostatic agent.	[4,26,27]
*S. aethiopis* L.	Habeş adaçayı, Yünlü adaçayı	Aerial parts, leaves, inflorescences, roots, infusion	Colds, flu, asthma, gastric spasms, stimulant, carminative, antiflatulent, reconstituent.	[4,13,24,28,29]
*S. dorystaechas* B.T.Drew	Dağ Çayı, Çalba çayı, Devren kekiği	Fresh/dried leaves, infusion	Treatment of colds and upper respiratory tract infections.	[24,30]
*S. tomentosa* Mill.	Şalba	Aerial parts, leaves, infusion	Gastrointestinal disorders, abdominal pain, colds, flu, and pharyngitis.	[4,31,32,33,34,35]
*S. glutinosa* L.	Oklu şalba, Yapışkan adaçayı	Aerial parts, leaves, flowers, infusion, decoction	Headaches, gastroenteritis, abdominal pain, throat infections, mouth sores, cough, sweating, burns, and wounds.	[36,37,38]

**Table 2:** Extraction yields, total phenolic contents, and antioxidant activities of the studied *Salvia* species.

Extracts	Yield (%) (*w/w*)	TPC (mg GAE/g Extract) ^1^	DPPH IC_50_ (mg/mL) ^2^
SD-I	14.71	111.50 ± 0.51 ^b^	0.09 ± 0.011 ^a^
ST-I	13.89	91.60 ± 0.66 ^c^	0.14 ± 0.024 ^b^
SA-I	12.64	40.75 ± 0.83 ^f^	0.35 ± 0.032 ^e,f^
SG-I	12.03	47.70 ± 0.96 ^e^	0.31 ± 0.037 ^e^
SE-I	11.93	74.30 ± 0.58 ^d^	0.16 ± 0.021 ^b^
SS-I	10.67	40.60 ± 0.95 ^f^	0.37 ± 0.016 ^g^
SD-E	3.62	125.55 ± 0.88 ^a^	0.07 ± 0.019 ^a^
ST-E	3.44	96.65 ± 0.70 ^c^	0.13 ± 0.026 ^b^
SG-E	3.25	68.45 ± 0.81 ^d^	0.26 ± 0.053 ^d^
SA-E	3.21	41.90 ± 0.30 ^f^	0.32 ± 0.028 ^e^
SE-E	3.19	71.45 ± 0.20 ^d^	0.18 ± 0.041 ^c^
SS-E	2.97	48.45 ± 0.73 ^e^	0.30 ± 0.034 ^e^
Gallic Acid	-	-	0.003 ± 0.0001 *

^1^: Values expressed as mg Gallic Acid Equivalents per gram of dry extract. ^2^: IC_50_ represents the concentration required to inhibit 50% of DPPH radicals. *: Positive control. Values are expressed as mean ± sd (*n* = 3). Different superscript letters (a–g) within the same column indicate statistically significant differences (*p* < 0.05) based on one-way ANOVA followed by Tukey’s post hoc test. SD: *S. dorystaechas*, ST: *S. tomentosa*, SA: *S. argentea*, SG: *S. glutinosa*, SE: *S. aethiopis*, SS: *S. sclarea*; I: Infusion, E: 70% Ethanol.

**Table 3:** Minimum Inhibitory Concentrations (MIC) of the studied *Salvia* extracts and reference drugs (μg/mL).

	*E. coli* ATCC 8739	*S. aureus* ATCC 6538	*C. tropicalis* ATCC 750	*C. albicans* ATCC 90028	*C.krusei* ATCC 14243
SD-I	1250	1250	625	5000	5000
SA-I	>2500	>2500	5000	>5000	>5000
ST-I	>2500	1250	5000	>5000	>5000
SS-I	>2500	>2500	5000	>5000	>5000
SE-I	>2500	>2500	5000	>5000	>5000
SG-I	>2500	>2500	2500	>5000	>5000
SG-E	2500	625	625	1250	1250
SD-E	2500	>2500	5000	625	625
SA-E	1250	1250	625	1250	1250
ST-E	1250	1250	625	1250	1250
SS-E	625	1250	625	2500	2500
SE-E	2500	2500	1250	2500	2500
Ciprofloxacin	0.25>	0.125	-	-	-
Fluconazole	-	-	2	64	64

SD: *S. dorystaechas*, ST: *S. tomentosa*, SA: *S. argentea*, SG: *S. glutinosa*, SE: *S. aethiopis*, SS: *S. sclarea*; I: Infusion, E: 70% Ethanol. Activity intensity classification for crude extracts: moderate activity (MIC ≤ 1000 µg/mL) is highlighted in [green]; weak activity (1000 < MIC ≤ 2500 µg/mL) is highlighted in [yellow]; very weak/Not active (MIC > 2500 µg/mL) is highlighted in [gray].

**Table 4:** IC_50_ values of selected extracts in NIH/3T3 cells after 24-h exposure.

Extracts	IC_50_ Values (µg/mL)	MIC Range (µg/mL)	SI_min_	SI_max_
SD-I	173.82 ± 25.09	625–5000	0.03	0.28
SD-E	110.06 ± 0.31	625–5000	0.02	0.18
ST-I	408.02 ± 4.63	1250– >5000	0.08	0.33
ST-E	562.37 ± 49.50	625–1250	0.45	0.90

Three independent experiments were performed, and IC_50_ values were calculated for each. Data are presented as mean IC_50_ ± sd (standard deviation). SD: *S. dorystaechas*, ST: *S. tomentosa*, I: Infusion, E: 70% Ethanol. IC_50_: Concentration required to reduce NIH/3T3 cell viability by 50%. SI_min_: Calculated using the highest MIC value obtained for each extract. SI_max_: Calculated using the lowest MIC value obtained for each extract.

**Table 5:** Bioactive constituents detected in various *Salvia* extracts by LC-HRMS analysis.

Compound	Class	Formula	Rt (min)	MW	Exp. (m/z) MS(+)	Exp. (m/z) MS(−)	Detected in Extracts
Luteolin	Flavonoid	C_15_H_10_O_6_	1.23	286.24	287.06	285.04	SG-I, SD-E, SA-I, SA-E, ST-I, ST-E, SS-I, SS-E, SE-I, SE-E
Luteolin-7-O-glucuronide	Flavonoid	C_21_H_18_O_12_	1.10	462.37	463.09	-	SG-I, SD-I, SD-E, SA-I, SA-E, ST-I, ST-E, SS-I, SS-E, SE-I
Rosmarinic acid	Phenolic Acid	C_18_H_16_O_8_	1.27	360.31	-	359.08	SG-I, SG-E
Rosmanol	Polyphenol	C_20_H_26_O_5_	2.72	346.42	347.19	345.17	SD-I, SD-E
Apigenin	Flavonoid	C_15_H_10_O_5_	1.41	270.24	271.06	269.05	SA-I, SA-E, ST-I, ST-E, SS-I, SS-E, SE-I
Acacetin	Flavonoid	C_16_H_12_O_5_	2.64	284.25	285.08	283.06	SA-E, SS-I, SS-E, SE-E
Cynaroside (Luteolin-7-O-glucoside)	Flavonoid	C_21_H_20_O_11_	1.09	448.38	449.11	447.09	ST-I, ST-E
Carnosol	Terpenoid	C_20_H_26_O_4_	2.72	330.4	331.19	329.18	SG-E, SD-E, SA-E, ST-I, ST-E, SS-I, SS-E
Carnosic acid	Terpenoid	C_20_H_28_O_4_	3.51	332.4	333.20	-	SD-E, ST-I, ST-E

Rt: Retention time; MW: Molecular weight; Exp.: Experimental. SD: *S. dorystaechas*, ST: *S. tomentosa*, SA: *S. argentea*, SG: *S. glutinosa*, SE: *S. aethiopis*, SS: *S. sclarea*; I: Infusion, E: 70% Ethanol.

## Data Availability

The original contributions presented in this study are included in the article/Supplementary Materials. Further inquiries can be directed to the corresponding author.

## Supplementary Materials

The following supporting information can be downloaded at: https://www.mdpi.com/article/10.3390/plants15111718/s1. Figure S1: Acacetin ESI (+) HRMS report, Figure S2: Acacetin ESI (−) HRMS report, Figure S3: Apigenin ESI (+) HRMS report, Figure S4: Apigenin ESI (−) HRMS report, Figure S5: Carnosic acid ESI (−) HRMS report, Figure S6: Carnosol ESI (+) HRMS report, Figure S7: Carnosol ESI (−) HRMS report, Figure S8: Cynaroside ESI (+) HRMS report, Figure S9: Cynaroside ESI (−) HRMS report, Figure S10: Luteolin ESI (+) HRMS report, Figure S11: Luteolin ESI (−) HRMS report, Figure S12: Luteolin-O- glucuronide ESI (+) HRMS report, Figure S13: Rosmanol ESI (+) HRMS report, Figure S14: Rosmanol ESI (−) HRMS report, Figure S15: Rosmarinic acid ESI (−) HRMS report.

## Abbreviations

The following abbreviations are used in this manuscript:

ABTS	2,2′-azino-bis (3-ethylbenzothiazoline-6-sulfonic acid)
ATCC	American Type Culture Collection
DPPH	2,2-diphenyl-1-picrylhydrazyl
CLSI	Clinical and Laboratory Standards Institute
DMSO	Dimethyl Sulfoxide
E	Ethanol
M	Methanol
mM	Millimolar
IC_50_	Half-maximal Inhibitory Concentration
MIC	Minimum Inhibitory Concentration
TPC	Total Phenol Content
SD	*S. dorystaechas*
sd	Standard Deviation
ST	*S. tomentosa*
SA	*S. argentea*
SE	*S. aethiopis*
SS	*S. sclerae*
I	Infusion

## References

[1] WFO: World Flora Online. https://www.worldfloraonline.org/.

[2] Celep F., Dirmenci T. (2017). Systematic and biogeographic overview of Lamiaceae in Turkey. Nat. Volatiles Essent. Oils.

[3] Nieto G. (2017). Biological activities of three essential oils of the Lamiaceae family. Medicines.

[4] Koyuncu O., Yaylaci O., Öztürk D., Erkara I.P., Savaroglu F., Akcoskun O., Ardic M. (2010). Risk categories and ethnobotanical features of the Lamiaceae taxa growing naturally in Osmaneli (Bilecik/Turkey) and environs. Biol. Divers. Conserv..

[5] Selvi S., Polat R., Çakılcıoğlu U., Celep F., Dirmenci T., Ertuğ F. (2022). An ethnobotanical review on medicinal plants of the Lamiaceae family in Turkey. Turk. J. Bot..

[6] Ertuğ F., Tümen G., Çelik A., Dirmenci T. (2004). Buldan (Denizli) Ethnobotanical Field Practice 2003. TUBA-KED.

[7] Ministry of Health of Turkey (2018). Turkish Pharmacopoeia (Türk Farmakopesi).

[8] BHMA (2009). British Herbal Pharmacopoeia (BHP).

[9] European Directorate for the Quality of Medicines & HealthCare (2013). European Pharmacopoeia (EP).

[10] World Health Organization (WHO) (1999). WHO Monographs on Selected Medicinal Plants.

[11] European Medicines Agency (EMA) Medicines for Use Outside of the EU. https://www.ema.europa.eu/en/human-regulatory-overview/marketing-authorisation/medicines-use-outside-european-union.

[12] European Scientific Cooperative on Phytotherapy (ESCOP) (2009). ESCOP Monographs.

[13] Davis P.H. (1982). Flora of Turkey and the East Aegean Islands.

[14] Baytop T. (1999). Therapy with Plants in Turkey (Past and Present).

[15] Karagözler A.A., Erdağ B., Emek Y.Ç., Uygun D.A. (2008). Antioxidant activity and proline content of leaf extracts from *Dorystoechas hastata*. Food Chem..

[16] Lu Y., Foo L.Y. (2002). Polyphenolics of *Salvia*—A review. Phytochemistry.

[17] Ozupek B., Pekacar S., Saltan N., Yıldız G., Gulcan Z., Orhan D.D., Kose Y.B. (2025). Assessment of the Enzyme Inhibitory Activities of *Salvia dorystaechas* and Its Phytochemical Analysis by RP-HPLC. Pharm. Chem. J..

[18] Ben Farhat M., Landoulsi A., Chaouch-Hamada R., Sotomayor J.A., Jordán M.J. (2013). Characterization and quantification of phenolic compounds and antioxidant properties of *Salvia* species growing in different habitats. Ind. Crops Prod..

[19] Tohma H., Köksal E., Kılıç Ö., Alan Y., Yılmaz M.A., Gülçin İ., Bursal E., Alwasel S.H. (2016). RP-HPLC/MS/MS Analysis of the Phenolic Compounds, Antioxidant and Antimicrobial Activities of *Salvia* L. Species. Antioxidants.

[20] Mocan A., Babotă M., Pop A., Fizeșan I., Diuzheva A., Locatelli M., Carradori S., Campestre C., Menghini L., Sisea C.R. (2020). Chemical Constituents and Biologic Activities of Sage Species: A Comparison between *Salvia officinalis* L., *S. glutinosa* L., and *S. transsylvanica (Schur ex Griseb. & Schenk) Schur*. Antioxidants.

[21] Tasheva K., Sulikovska I., Georgieva A., Djeliova V., Lozanova V., Vasileva A., Ivanov I., Denev P., Lazarova M., Vassileva V. (2025). Phytochemical Profile, Antioxidant Capacity and Anticancer Potential of Water Extracts from In Vitro Cultivated *Salvia aethiopis*. Molecules.

[22] Dilgen M.N., Aydın B., Vural N., Koç M. (2025). Analysis of phytochemical profiles of different extracts of *Salvia* (*S. sclarea*, *S. huberi*, *S. fruticosa* and *S. aethiopis*) by HPLC-PDA, evaluation of their antioxidant capacity and antimicrobial activity. Chem. Biodivers..

[23] Kaya M.M., Tutun H. (2025). Optimization of microwave-assisted extraction to maximize phenolic content and antioxidant capacity of *Salvia tomentosa* using response surface methodology. Medit. Vet. J..

[24] TÜBİVES: Turkish Plants Data Service. http://www.tubives.net/.

[25] Altindag E., Ozturk M. (2011). Ethnomedicinal studies on the plant resources of East Anatolia, Turkey. Procedia Soc. Behav. Sci..

[26] Benabdesslem Y., Hachem K., Kahloula K., Slimani M. (2017). Ethnobotanical Survey, Preliminary Physico-Chemical and Phytochemical Screening of *Salvia argentea* (L.) Used by Herbalists of the Saïda Province in Algeria. Plants.

[27] Benabdesslem Y., Hachem K., Kahloula K., Slimani M. (2018). An ethnopharmacological study of *Salvia argentea* used by the local people of Saida in northwestern Algeria. South Asian J. Exp. Biol..

[28] Poyraz İ.E., Çiftçi Akalin G., Öztürk N. (2017). Phenolic contents, in vitro antioxidant and cytotoxicity activities of *Salvia aethiopis* L. and *S. ceratophylla* L.. Rec. Nat. Prod..

[29] Kostić M., Miladinović B., Milutinović M., Branković S., Živanović S., Zlatković B., Kitić D. (2017). Rosmarinic and caffeic acid content and antioxidant potential of the *Salvia aethiopis* L. extracts. Acta Med. Median.

[30] Özcan M.M., Chalchat J.C., Figueredo G., Bagci Y., Dural H., Savran A., Al-Juhaimi F.Y. (2016). Chemical composition of the essential oil of the flowers and leaves of Çalba tea (*Dorystoechas hastata* Boiss & Helder. ex Bentham). J. Essent. Oil Bear. Plants.

[31] Bulut G.E., Tuzlacı E. (2011). Folk medicinal plants of Bayramiç (Çanakkale-Turkey). J. Fac. Pharm. Istanb. Univ..

[32] Fakir H., Korkmaz M., Icel B. (2016). Medicinal plants traditionally used for pain alleviation in Antalya Province, Turkey. Stud. Ethno-Med..

[33] Karaköse M., Akbulut S., Özkan Z.C. (2019). Ethnobotanical study of medicinal plants in Torul District, Turkey. Bangladesh J. Plant Taxon..

[34] Polat R., Satıl F. (2012). An ethnobotanical survey of medicinal plants in Edremit Gulf (Balıkesir-Turkey). J. Ethnopharmacol..

[35] Tuzlacı E., Erol M.K. (1999). Turkish folk medicinal plants, Part II: Eğirdir (Isparta). Fitoterapia.

[36] Saraç D.U., Özkan Z.C., Akbulut S. (2013). Ethnobotanic features of Rize/Turkey province. Biol. Divers. Conserv..

[37] Nicolescu A., Babotă M., Ilea M., Dias M.I., Calhelha R.C., Gavrilaș L., Rocchetti G., Crișan G., Mocan A., Barros L. (2022). Potential therapeutic applications of infusions and hydroalcoholic extracts of Romanian glutinous sage (*Salvia glutinosa* L.). Front. Pharmacol..

[38] Idolo M., Motti R., Mazzoleni S. (2010). Ethnobotanical and phytomedicinal knowledge in a long-history protected area, the Abruzzo, Lazio and Molise National Park (Italian Apennines). J. Ethnopharmacol..

[39] Todaro G.J., Green H. (1963). Quantitative studies of the growth of mouse embryo cells in culture and their development into established lines. J. Cell Biol..

[40] Criollo-Mendoza M.S., Ramos-Payán R., Contreras-Angulo L.A., Gutiérrez-Grijalva E.P., León-Félix J., Villicaña C., Angulo-Escalante M.A., Heredia J.B. (2022). Cytotoxic Activity of Polyphenol Extracts from Three Oregano Species. Molecules.

[41] Gonzalez-Pastor R., Carrera-Pacheco S.E., Zúñiga-Miranda J., Rodríguez-Pólit C., Mayorga-Ramos A., Guamán L.P., Barba-Ostria C. (2023). Current Landscape of Methods to Evaluate Antimicrobial Activity of Natural Extracts. Molecules.

[42] Zouine N., El Ghachtouli N., El Abed S., Koraichi S.I. (2024). A comprehensive review on medicinal plant extracts as antibacterial agents: Factors, mechanism insights and future prospects. Sci. Afr..

[43] Reviana R., Usman A.N., Raya I., Aliyah, Dirpan A., Arsyad A., Fendi F. (2021). Analysis of antioxidant activity on cocktail honey products as female pre-conception supplements. Gac. Sanit..

[44] Belete B.B., Ozkan J., Kalaiselvan P., Willcox M. (2025). Clinical Potential of Essential Oils: Cytotoxicity, Selectivity Index, and Efficacy for Combating Gram-Positive ESKAPE Pathogens. Molecules.

[45] Mervić M., Bival Štefan M., Kindl M., Blažeković B., Marijan M., Vladimir-Knežević S. (2022). Comparative Antioxidant, Anti-Acetylcholinesterase and Anti-α-Glucosidase Activities of Mediterranean *Salvia* Species. Plants.

[46] Hanganu D., Olah N.K., Pop C.E., Vlase L., Oniga I., Ciocarlan N., Matei A., Pușcaș C., Silaghi-Dumitrescu R., Benedec D. (2019). Evaluation of polyphenolic profile and antioxidant activity for some *Salvia* species. Farmacia.

[47] Caylak E. (2024). Determination of Antioxidant and Anti-Inflammatory Properties of *Salvia aethiopis*/*sclarea* and Synthesized Silver Nanoparticles. Ann. Med. Res..

[48] İskender N., Çoban N. (2025). Antimicrobial Activity and Bioactive Compound Profile of Extracts from *Tanacetum Parthenium* and *Salvia Glutinosa* Naturally Growing in Artvin Province, Türkiye. C. R. Acad. Bulg. Sci..

[49] Sultana B., Anwar F., Ashraf M. (2009). Effect of extraction solvent/technique on the antioxidant activity of selected medicinal plant extracts. Molecules.

[50] Alhamedi A., Demiroz Akbulut T., Baykan S., Gümüştaş B., Sanci E., Alsakini K.A.M.H., Nalbantsoy A., Buhur A., Yavasoğlu A., Karabay Yavasoğlu N.Ü. (2025). *Salvia argentea* L. extract inhibits the production of NO, and pro-inflammatory cytokines (IL-1β, IL-6, and TNF-α), alleviates the inflammatory response of LPS-induced macrophages cells, and reduces the CRP level on carrageenan-induced paw edema. Inflammopharmacology.

[51] Balkir S., Hazman Ö., Aksoy L., Yılmaz M.A., Cakir O., Erol İ. (2023). Phytochemical Profile, Antioxidant and Antimicrobial Potency of Aerial Parts of *Salvia Tomentosa* Miller. Acta Chim. Slov..

[52] Dinçer C., Tontul İ., Çam İ.B., Özdemir K.S., Topuz A., Nadeem H.Ş., Ay S.T., Göktürk R.S. (2013). Phenolic composition and antioxidant activity of *Salvia tomentosa* Miller: Effects of cultivation, harvesting year, and storage. Turk. J. Agric. For..

[53] Zhang T., Qiu Y., Luo Q., Zhao L., Yan X., Ding Q., Jiang H., Yang H. (2018). The mechanism by which luteolin disrupts the cytoplasmic membrane of methicillin-resistant *Staphylococcus aureus*. J. Phys. Chem. B.

[54] Ivanov M., Kostić M., Stojković D., Soković M. (2022). Rosmarinic acid–modes of antimicrobial and antibiofilm activities of a common plant polyphenol. S. Afr. J. Bot..

[55] Ojeda-Sana A.M., Repetto V., Moreno S. (2013). Carnosic acid is an efflux pumps modulator by dissipation of the membrane potential in *Enterococcus faecalis* and *Staphylococcus aureus*. World J. Microbiol. Biotechnol..

[56] Piątczak E., Kolniak-Ostek J., Gonciarz W., Lisiecki P., Kalinowska-Lis U., Szemraj M., Chmiela M., Zielińska S. (2024). The Effect of *Salvia tomentosa* Miller Extracts, Rich in Rosmarinic, Salvianolic and Lithospermic Acids, on Bacteria Causing Opportunistic Infections. Molecules.

[57] Afzal T., Proćków J., Łyczko J. (2025). Bioactive chemical composition and pharmacological insights into *Salvia* species. Front. Mol. Biosci..

[58] Yazıcı-Tütüniş S., Alim-Toraman G.Ö., Dincel E.D., Tufan S., Akalın E., Tan E., Ulusoy-Güzeldemirci N., Gören A.C., Miski M., Topçu G. (2025). In silico and in vitro antibacterial evaluation of eight Anatolian *Salvia* species with chemical profiling by LC-HRMS. Sci. Rep..

[59] Jan S., Khaliq T., Sultan P., Hassan Q.P., Gupta S., Ahmed Z. (2026). *Salvia sclarea*: A comprehensive review of its pharmacology, phytochemistry, ethnobotany and traditional uses. Curr. Plant Biol..

[60] Jassbi A.R., Zare S., Firuzi O., Xiao J. (2016). Bioactive phytochemicals from shoots and roots of *Salvia* species. Phytochem. Rev..

[61] Tavan M., Azizi A., Sarikhani H., Mirjalili M.H., Rigano M.M. (2022). Phenolics diversity among wild populations of *Salvia multicaulis*: As a precious source for antimicrobial and antioxidant applications. Nat. Prod. Res..

[62] Kaplan O., Saltan N., Köse A., Köse Y.B., Çelebier M. (2023). A Plant Metabolomic Approach to Identify the Difference of the Seeds and Flowers Extracts of *Carthamus tinctorius* L.. Mass Spectrom. Lett..

[63] Singleton V.L., Orthofer R., Lamuela-Raventos R.M. (1999). Analysis of total phenols and other oxidation substrates and antioxidants by means of folin-ciocalteu reagent. Methods in Enzymology.

[64] Kumarasamy Y., Byres M., Cox P.J., Jaspars M., Nahar L., Sarker S.D. (2007). Screening seeds of some Scottish plants for free radical scavenging activity. Phytother. Res..

[65] (2006). Methods for Dilution Antimicrobial Susceptibility Tests for Bacteria That Grow Aerobically.

[66] (2002). Reference Method for Broth Dilution Antifungal Susceptibility Testing of Yeasts.

[67] Riss T.L., Moravec R.A., Niles A.L., Duellman S., Benink H.A., Worzella T.J., Minor L., Markossian S., Grossman A., Baskir H. (2004). Cell Viability Assays. Assay Guidance Manual.

[68] Baysal M., Atlı-Eklioğlu Ö. (2021). Comparison of the toxicity of pure compounds and commercial formulations of imidacloprid and acetamiprid on HT-29 cells: Single and mixture exposure. Food Chem. Toxicol..

