# Supplementary material for: Evaluation of the Therapeutic Potential and Safety Profile of Six Salvia Species Native to Türkiye
Source: Plants (Basel). 2026 Jun 2;15(11):1718. doi: 10.3390/plants15111718 (PMC13259035; doi:10.3390/plants15111718)
Supplement: Supplementary file 1 [file plants-15-01718-s001.zip › plants-4313886-supplementary.pdf]

Data File: C:\LabSolutions\Data\Analiz\BETÜL AYDIN\NS-36-E_19.lcd

Elmt	Val.	Min	Max	Elmt	Val.	Min	Max	Elmt	Val.	Min	Max	Elmt	Val.	Min	Max	Use Adduct
H	1	10	36	O	2	0	15	S	2	0	0	Ru	2	0	0	H
C	4	10	28	F	1	0	0	Cl	1	0	0	Pd	2	0	0	
N	3	0	0	P	3	0	0	Br	1	0	0	I	3	0	0	

Error Margin (ppm):	5	DBE Range:	0.0 - 25.0	Electron Ions:	both
HC Ratio:	unlimited	Apply N Rule:	yes	Use MSn Info:	yes
Max Isotopes:	5	Isotope RI (%):	1.00	Isotope Res:	9000
MSn Iso RI (%):	10.00	MSn Logic Mode:	AND	Max Results:	50

Event#: 1 MS(E+) Ret. Time : 2.653 -> 2.653 Scan# : 399 -> 399

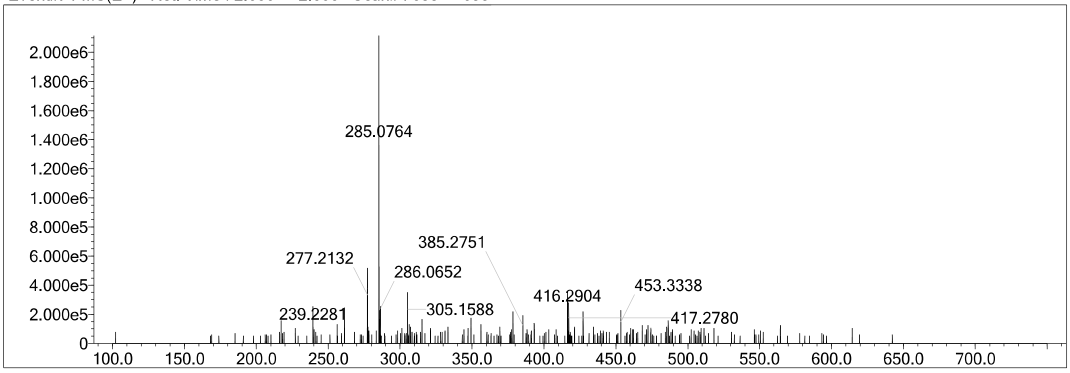

Measured region for 285.0764 m/z

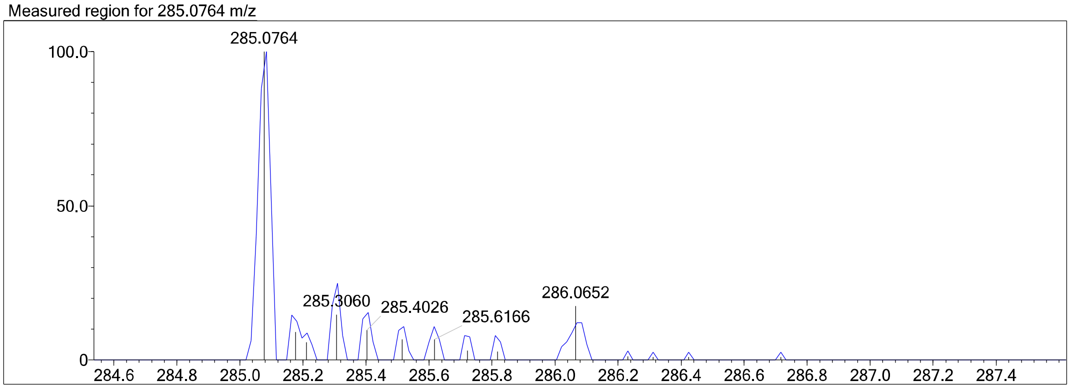

C16 H12 O5 [M+H]+ : Predicted region for 285.0758 m/z

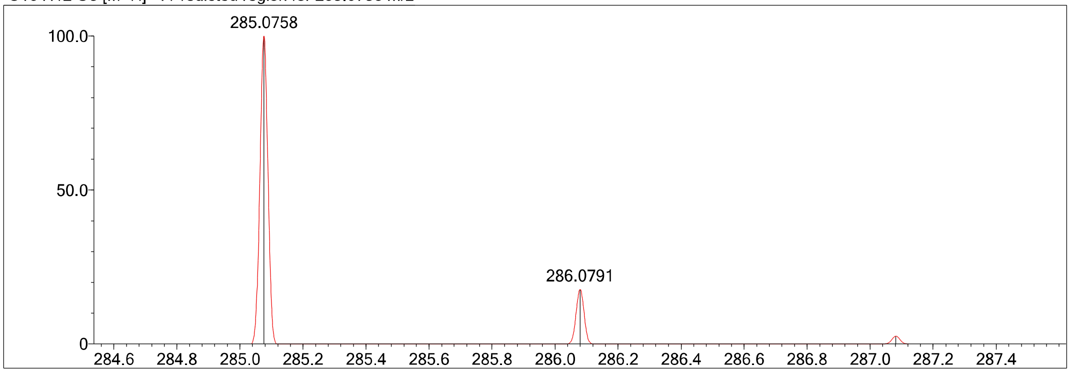

Rank	Score	Formula (M)	Ion	Meas. m/z	Pred. m/z	Df. (mDa)	Df. (ppm)	Iso	DBE
1	45.95	C16 H12 O5	[M+H]+	285.0764	285.0758	0.6	2.10	47.25	11.0

Figure S1. Acacetin ESI (+) HRMS report

Data File: C:\LabSolutions\Data\Analiz\BETÜL AYDIN\NS-36-E_19.lcd

Elmt	Val.	Min	Max	Elmt	Val.	Min	Max	Elmt	Val.	Min	Max	Elmt	Val.	Min	Max	Use Adduct
H	1	10	36	O	2	0	15	S	2	0	0	Ru	2	0	0	H
C	4	10	28	F	1	0	0	Cl	1	0	0	Pd	2	0	0	
N	3	0	0	P	3	0	0	Br	1	0	0	I	3	0	0	

Error Margin (ppm):	5	DBE Range:	0.0 - 25.0	Electron Ions:	both
HC Ratio:	unlimited	Apply N Rule:	yes	Use MSn Info:	yes
Max Isotopes:	5	Isotope RI (%):	1.00	Isotope Res:	9000
MSn Iso RI (%):	10.00	MSn Logic Mode:	AND	Max Results:	50

Event#: 2 MS(E-) Ret. Time : 2.587 -> 2.707 - 8.240 -> 11.455 Scan# : 390 -> 408 - 1238 -> 1720

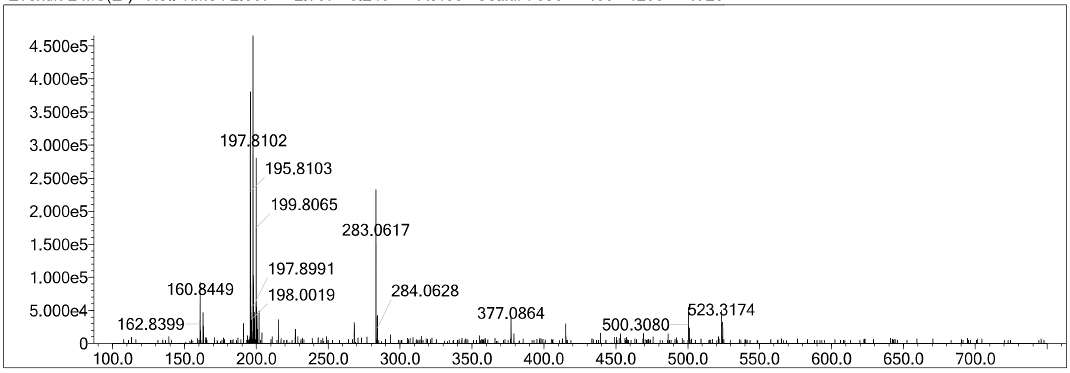

Measured region for 283.0617 m/z

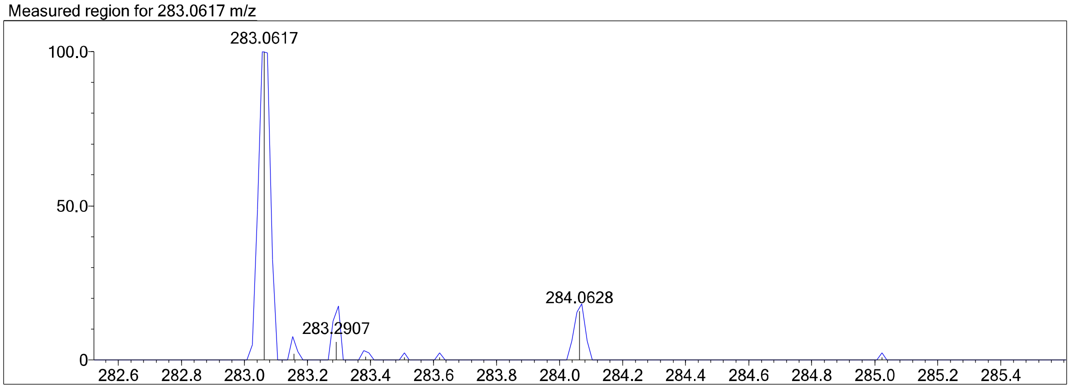

C16 H12 O5 [M-H]- : Predicted region for 283.0612 m/z

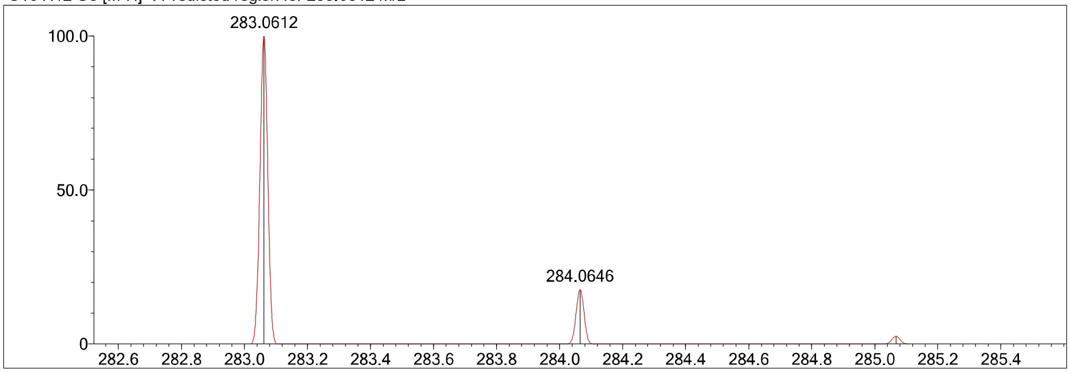

Rank	Score	Formula (M)	Ion	Meas. m/z	Pred. m/z	Df. (mDa)	Df. (ppm)	Iso	DBE
1	98.08	C16 H12 O5	[M-H]-	283.0617	283.0612	0.5	1.77	100.00	11.0

Figure S2. Acacetin ESI (−) HRMS report

Data File: C:\LabSolutions\Data\Analiz\BETÜL AYDIN\NS-35-I_16.lcd

Elmt	Val.	Min	Max	Elmt	Val.	Min	Max	Elmt	Val.	Min	Max	Elmt	Val.	Min	Max	Use Adduct
H	1	10	36	O	2	0	15	S	2	0	0	Ru	2	0	0	H
C	4	10	28	F	1	0	0	Cl	1	0	0	Pd	2	0	0	
N	3	0	0	P	3	0	0	Br	1	0	0	I	3	0	0	

Error Margin (ppm):	5	DBE Range:	0.0 - 25.0	Electron Ions:	both
HC Ratio:	unlimited	Apply N Rule:	yes	Use MSn Info:	yes
Max Isotopes:	5	Isotope RI (%):	1.00	Isotope Res:	9000
MSn Iso RI (%):	10.00	MSn Logic Mode:	AND	Max Results:	50

Event#: 1 MS(E+) Ret. Time : 1.427 -> 1.427 Scan# : 215 -> 215

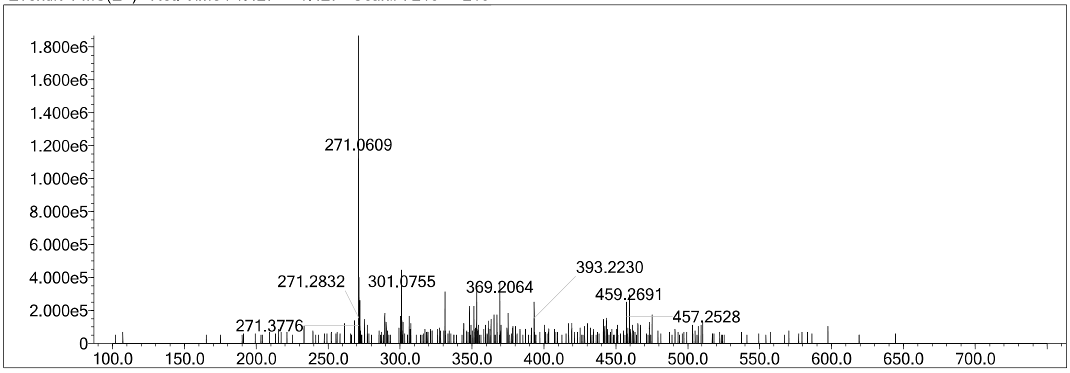

Measured region for 271.0609 m/z

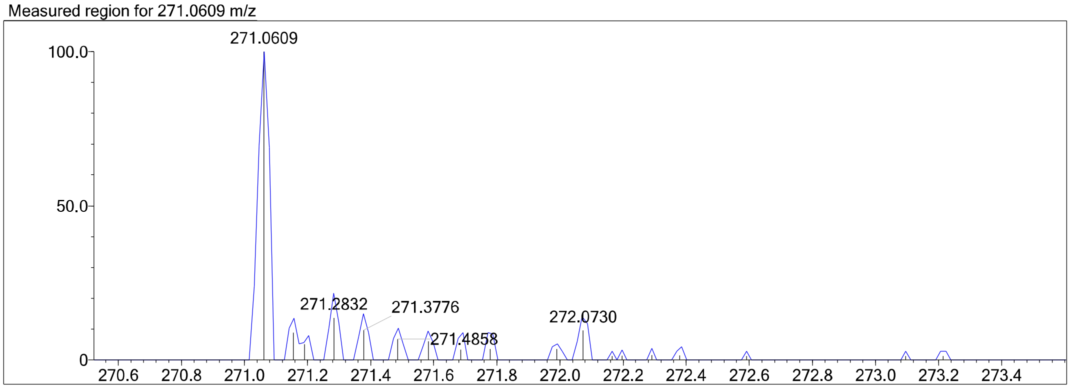

C15 H10 O5 [M+H]+ : Predicted region for 271.0601 m/z

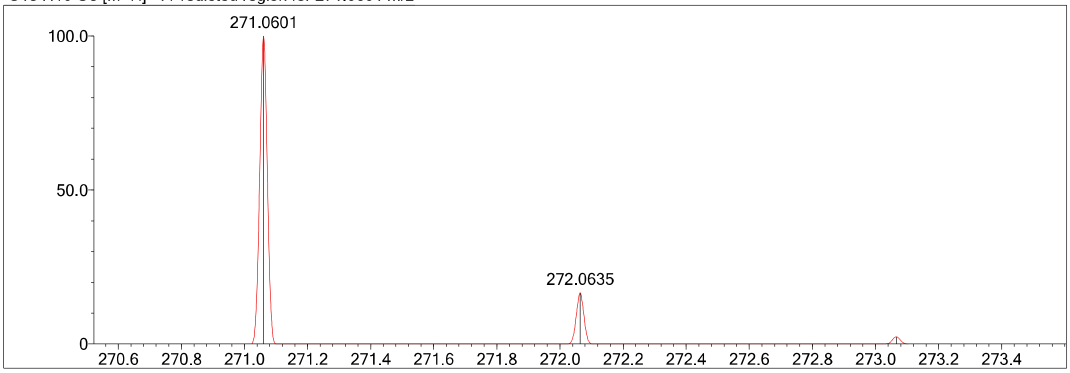

Rank	Score	Formula (M)	Ion	Meas. m/z	Pred. m/z	Df. (mDa)	Df. (ppm)	Iso	DBE
1	40.75	C15 H10 O5	[M+H]+	271.0609	271.0601	0.8	2.95	42.84	11.0

Figure S3. Apigenin ESI (+) HRMS report

Data File: C:\LabSolutions\Data\Analiz\BETÜL AYDIN\NS-35-I_16.lcd

Elmt	Val.	Min	Max	Elmt	Val.	Min	Max	Elmt	Val.	Min	Max	Elmt	Val.	Min	Max	Use Adduct
H	1	10	36	O	2	0	15	S	2	0	0	Ru	2	0	0	H
C	4	10	28	F	1	0	0	Cl	1	0	0	Pd	2	0	0	
N	3	0	0	P	3	0	0	Br	1	0	0	I	3	0	0	

Error Margin (ppm):	5	DBE Range:	0.0 - 25.0	Electron Ions:	both
HC Ratio:	unlimited	Apply N Rule:	yes	Use MSn Info:	yes
Max Isotopes:	5	Isotope RI (%):	1.00	Isotope Res:	9000
MSn Iso RI (%):	10.00	MSn Logic Mode:	AND	Max Results:	50

Event#: 2 MS(E-) Ret. Time : 1.400 -> 1.400 Scan# : 212 -> 212

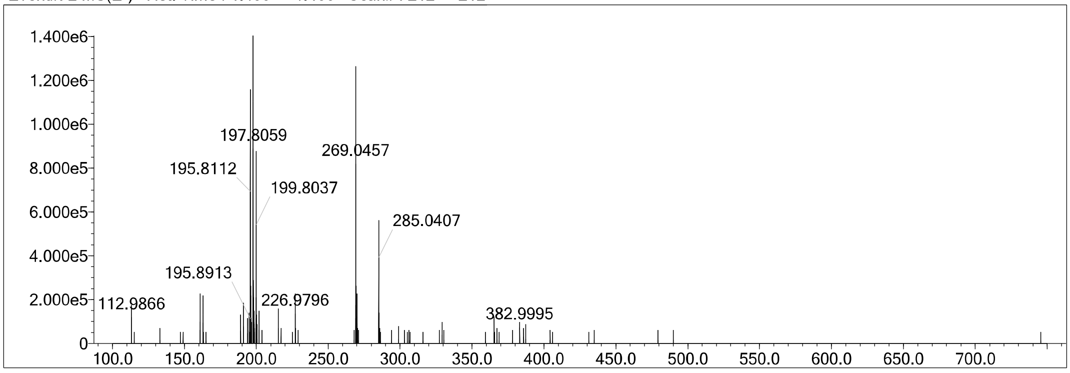

Measured region for 269.0457 m/z

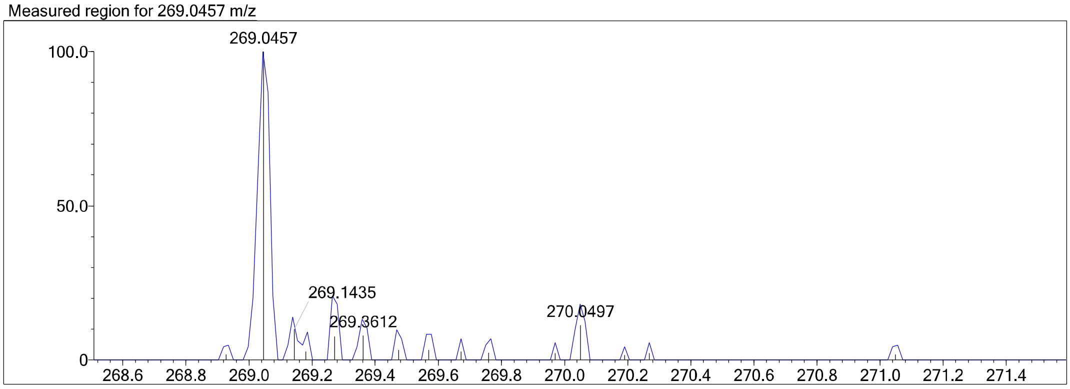

C15 H10 O5 [M-H]- : Predicted region for 269.0455 m/z

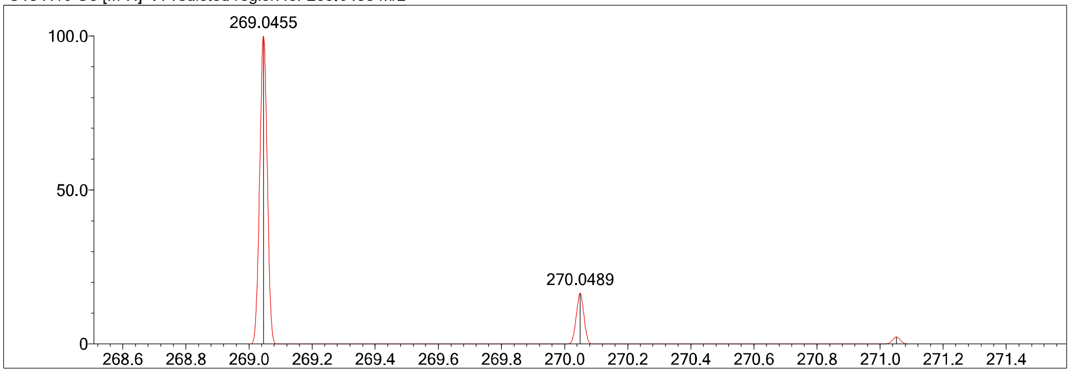

Rank	Score	Formula (M)	Ion	Meas. m/z	Pred. m/z	Df. (mDa)	Df. (ppm)	Iso	DBE
1	87.47	C15 H10 O5	[M-H]-	269.0457	269.0455	0.2	0.74	87.47	11.0

Figure S4. Apigenin ESI (−) HRMS report

Formula Predictor Report - NS-34-E_15.lcd

Data File: C:\LabSolutions\Data\Analiz\BETÜL AYDIN\NS-34-E_15.lcd

Elmt	Val.	Min	Max	Elmt	Val.	Min	Max	Elmt	Val.	Min	Max	Elmt	Val.	Min	Max	Use Adduct
H	1	10	36	O	2	0	15	S	2	0	0	Ru	2	0	0	H
C	4	10	30	F	1	0	0	Cl	1	0	0	Pd	2	0	0	
N	3	0	0	P	3	0	0	Br	1	0	0	I	3	0	0	

Error Margin (ppm):	5	DBE Range:	0.0 - 25.0	Electron Ions:	both
HC Ratio:	unlimited	Apply N Rule:	yes	Use MSn Info:	yes
Max Isotopes:	5	Isotope RI (%):	1.00	Isotope Res:	9000
MSn Iso RI (%):	10.00	MSn Logic Mode:	AND	Max Results:	50

Event#: 1 MS(E+) Ret. Time : 2.587 -> 2.893 - 5.840 -> 8.422 Scan# : 389 -> 435 - 877 -> 1265

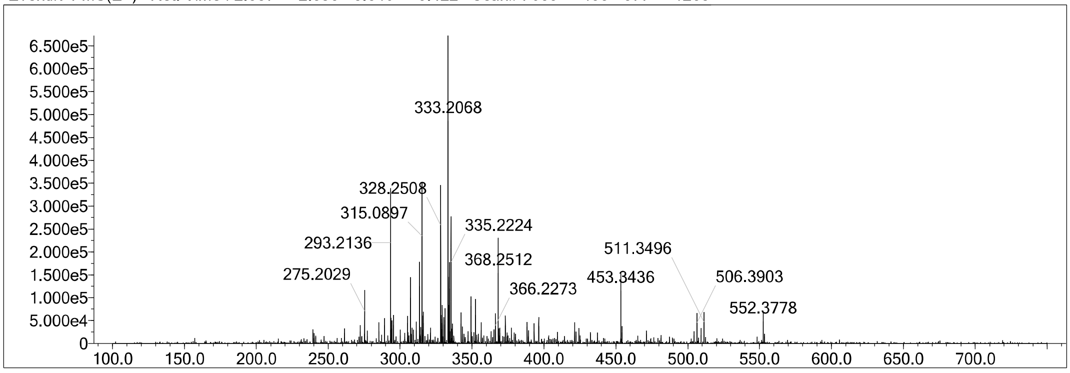

Measured region for 333.2068 m/z

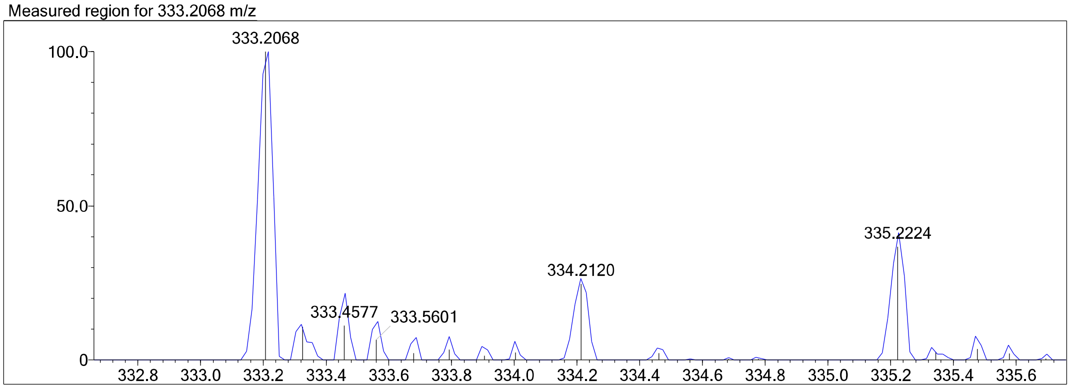

C20 H28 O4 [M+H]+ : Predicted region for 333.2060 m/z

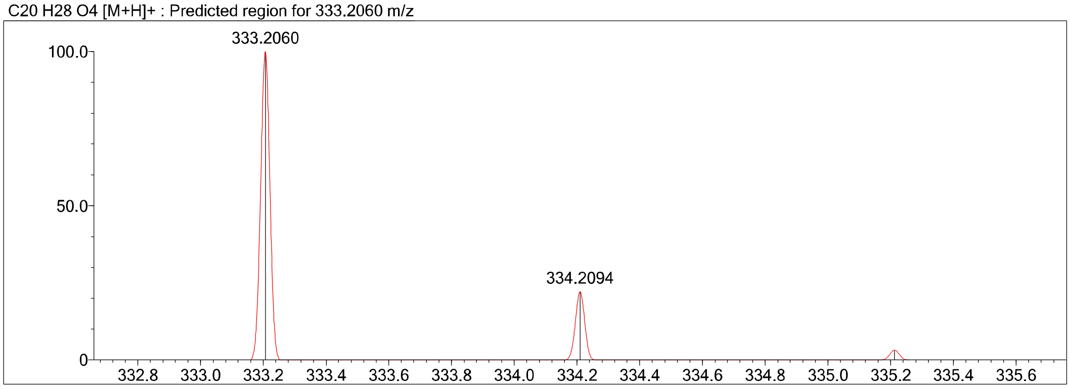

Rank	Score	Formula (M)	Ion	Meas. m/z	Pred. m/z	Df. (mDa)	Df. (ppm)	Iso	DBE
1	29.68	C20 H28 O4	[M+H]+	333.2068	333.2060	0.8	2.40	30.75	7.0

Figure S5. Carnosic acid ESI (−) HRMS report

Formula Predictor Report - NS-31-E_11.lcd

Data File: C:\LabSolutions\Data\Analiz\BETÜL AYDIN\NS-31-E_11.lcd

Elmt	Val.	Min	Max	Elmt	Val.	Min	Max	Elmt	Val.	Min	Max	Elmt	Val.	Min	Max	Use Adduct
H	1	10	36	O	2	0	15	S	2	0	0	Ru	2	0	0	H
C	4	10	30	F	1	0	0	Cl	1	0	0	Pd	2	0	0	
N	3	0	0	P	3	0	0	Br	1	0	0	I	3	0	0	

Error Margin (ppm): 5
HC Ratio: unlimited
Max Isotopes: 5
MSn Iso RI (%): 10.00

DBE Range: 0.0 - 25.0
Apply N Rule: yes
Isotope RI (%): 1.00
MSn Logic Mode: AND

Electron Ions: both
Use MSn Info: yes
Isotope Res: 9000
Max Results: 50

Event#: 1 MS(E+) Ret. Time : 6.400 -> 6.933 Scan# : 961 -> 1041

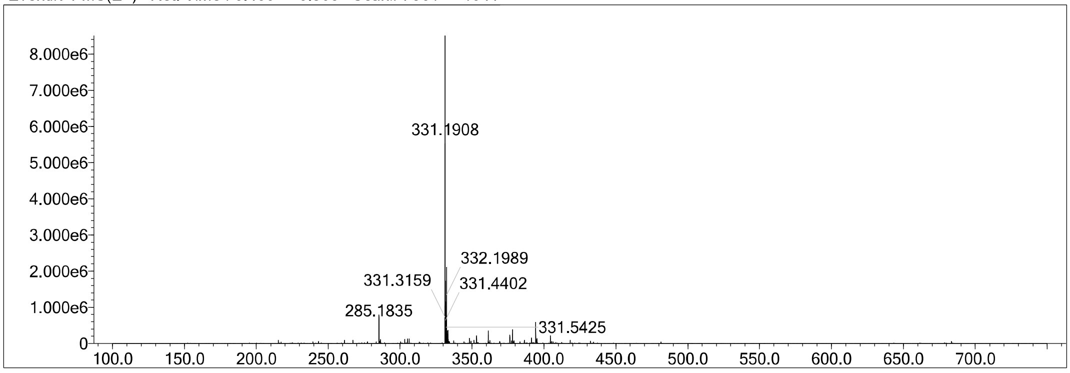

Measured region for 331.1908 m/z

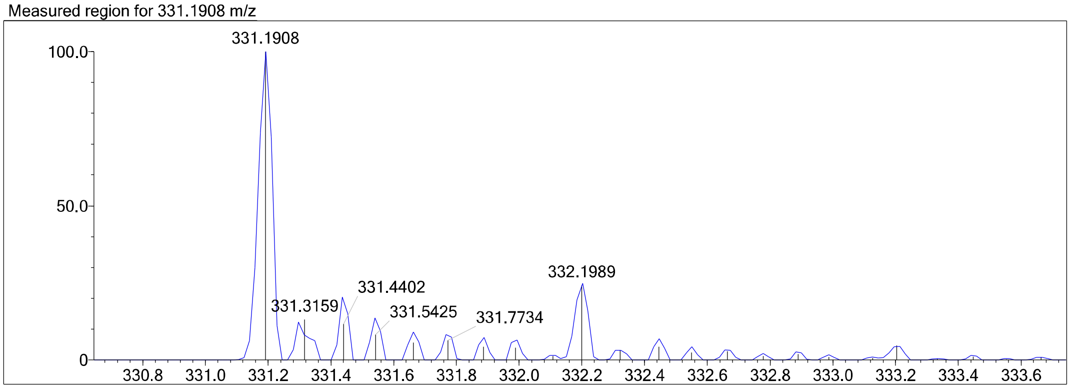

C20 H26 O4 [M+H]+ : Predicted region for 331.1904 m/z

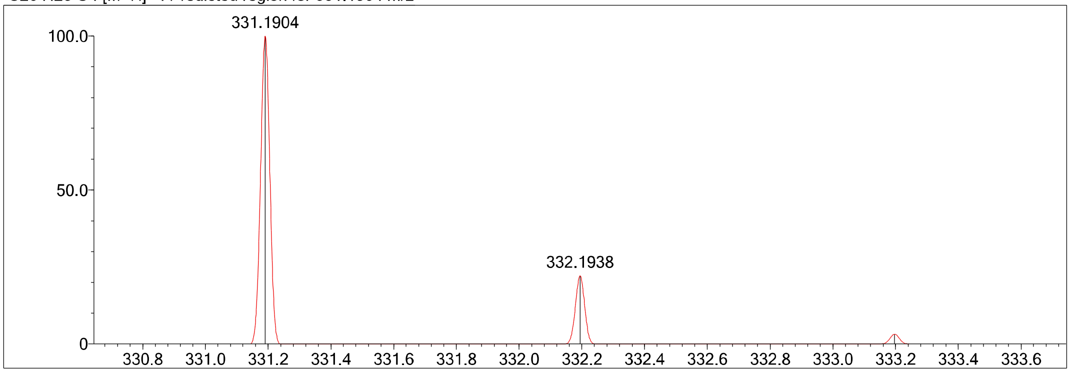

Rank	Score	Formula (M)	Ion	Meas. m/z	Pred. m/z	Df. (mDa)	Df. (ppm)	Iso	DBE
1	77.88	C20 H26 O4	[M+H]+	331.1908	331.1904	0.4	1.21	78.29	8.0

Figure S6. Carnosol ESI (+) HRMS report

Data File: C:\LabSolutions\Data\Analiz\BETÜL AYDIN\NS-31-E_11.lcd

Elmt	Val.	Min	Max	Elmt	Val.	Min	Max	Elmt	Val.	Min	Max	Elmt	Val.	Min	Max	Use Adduct
H	1	10	36	O	2	0	15	S	2	0	0	Ru	2	0	0	H
C	4	10	30	F	1	0	0	Cl	1	0	0	Pd	2	0	0	
N	3	0	0	P	3	0	0	Br	1	0	0	I	3	0	0	

Error Margin (ppm):	5	DBE Range:	0.0 - 25.0	Electron Ions:	both
HC Ratio:	unlimited	Apply N Rule:	yes	Use MSn Info:	yes
Max Isotopes:	5	Isotope RI (%):	1.00	Isotope Res:	9000
MSn Iso RI (%):	10.00	MSn Logic Mode:	AND	Max Results:	50

Event#: 2 MS(E-) Ret. Time : 6.400 -> 6.893 - 8.880 -> 11.515 Scan# : 962 -> 1036 - 1334 -> 1730

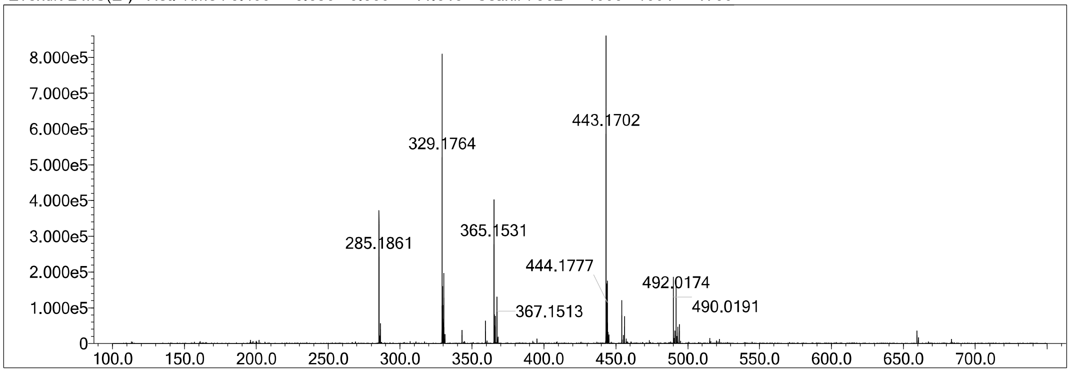

Measured region for 329.1764 m/z

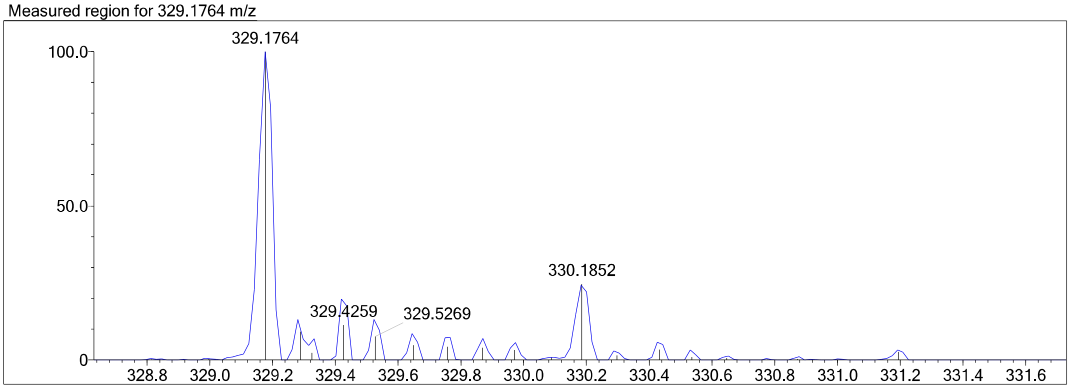

C20 H26 O4 [M-H]- : Predicted region for 329.1758 m/z

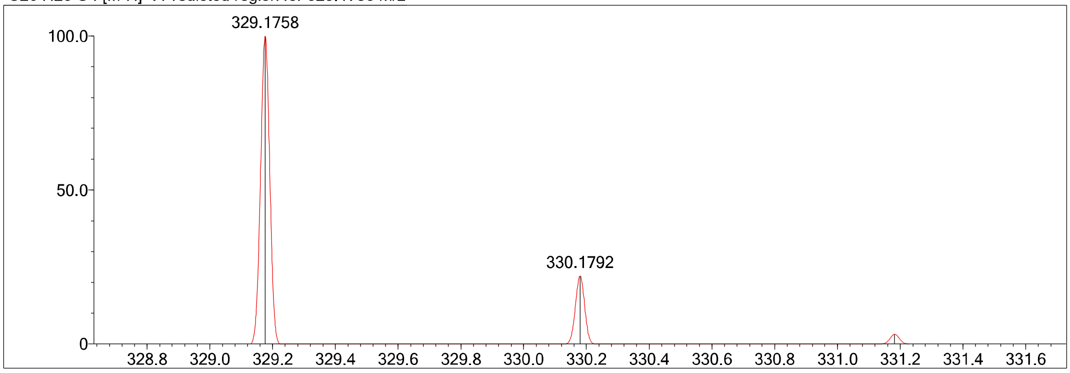

Rank	Score	Formula (M)	Ion	Meas. m/z	Pred. m/z	Df. (mDa)	Df. (ppm)	Iso	DBE
1	73.27	C20 H26 O4	[M-H]-	329.1764	329.1758	0.6	1.82	74.80	8.0

Figure S7. Carnosol ESI (−) HRMS report

Formula Predictor Report - NS-34-E_15.lcd Page 1 of 1

Data File: C:\LabSolutions\Data\Analiz\BETÜL AYDIN\NS-34-E_15.lcd

Elmt	Val.	Min	Max	Elmt	Val.	Min	Max	Elmt	Val.	Min	Max	Elmt	Val.	Min	Max	Use Adduct
H	1	10	36	O	2	0	15	S	2	0	0	Ru	2	0	0	H
C	4	10	28	F	1	0	0	Cl	1	0	0	Pd	2	0	0	
N	3	0	0	P	3	0	0	Br	1	0	0	I	3	0	0	

Error Margin (ppm):	5	DBE Range:	0.0 - 25.0	Electron Ions:	both
HC Ratio:	unlimited	Apply N Rule:	yes	Use MSn Info:	yes
Max Isotopes:	5	Isotope RI (%):	1.00	Isotope Res:	9000
MSn Iso RI (%):	10.00	MSn Logic Mode:	AND	Max Results:	50

Event#: 1 MS(E+) Ret. Time : 1.053 -> 1.160 Scan# : 159 -> 175

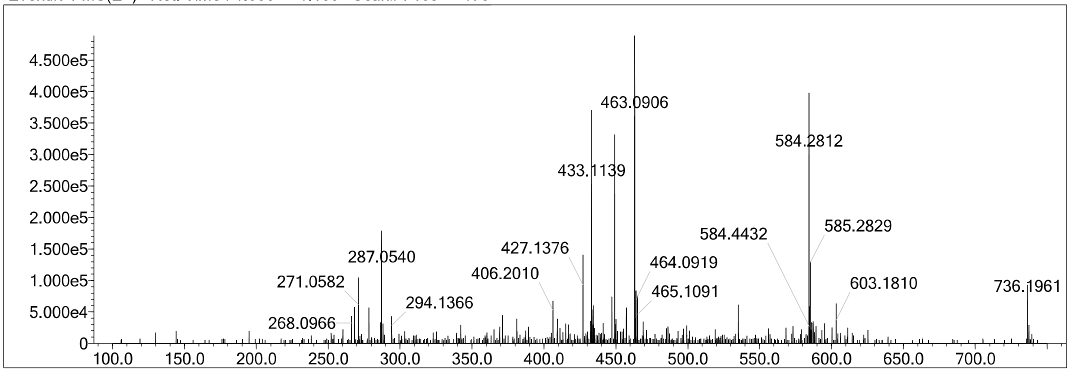

Measured region for 449.1086 m/z

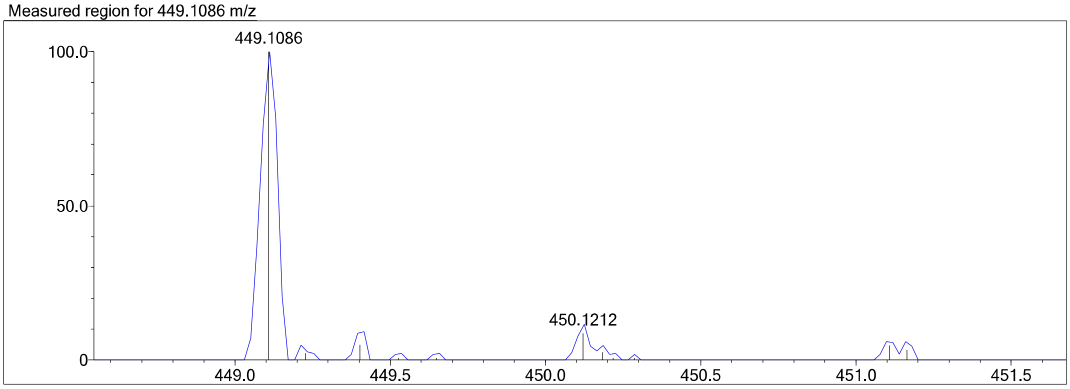

C21 H20 O11 [M+H]+ : Predicted region for 449.1078 m/z

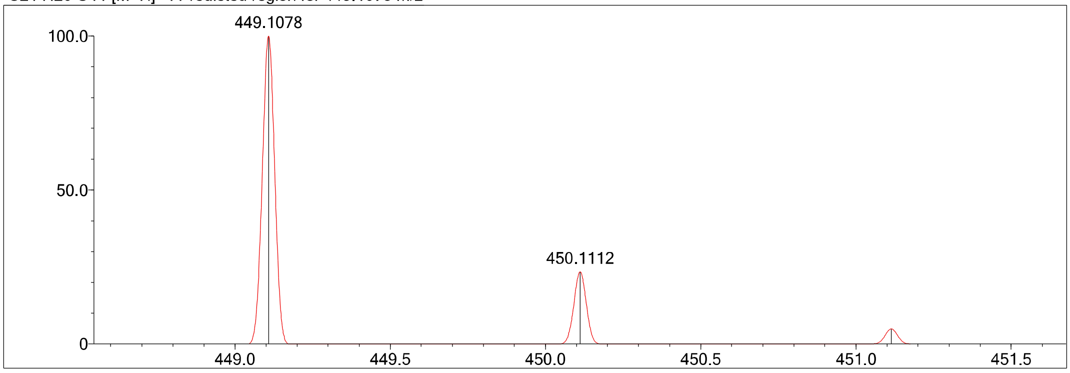

Rank	Score	Formula (M)	Ion	Meas. m/z	Pred. m/z	Df. (mDa)	Df. (ppm)	Iso	DBE
1	44.31	C21 H20 O11	[M+H]+	449.1086	449.1078	0.8	1.78	45.19	12.0

Figure S8. Cynaroside ESI (+) HRMS report

Data File: C:\LabSolutions\Data\Analiz\BETÜL AYDIN\NS-34-E_15.lcd

Elmt	Val.	Min	Max	Elmt	Val.	Min	Max	Elmt	Val.	Min	Max	Elmt	Val.	Min	Max	Use Adduct
H	1	10	36	O	2	0	15	S	2	0	0	Ru	2	0	0	H
C	4	10	28	F	1	0	0	Cl	1	0	0	Pd	2	0	0	
N	3	0	0	P	3	0	0	Br	1	0	0	I	3	0	0	

Error Margin (ppm):	5	DBE Range:	0.0 - 25.0	Electron Ions:	both
HC Ratio:	unlimited	Apply N Rule:	yes	Use MSn Info:	yes
Max Isotopes:	5	Isotope RI (%):	1.00	Isotope Res:	9000
MSn Iso RI (%):	10.00	MSn Logic Mode:	AND	Max Results:	50

Event#: 2 MS(E-) Ret. Time : 0.947 -> 1.053 - 9.093 -> 11.348 Scan# : 144 -> 160 - 1366 -> 1704

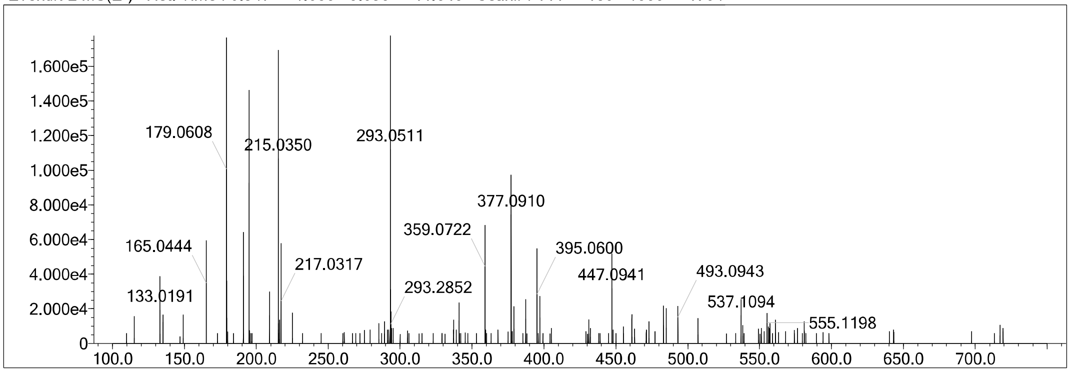

Measured region for 447.0941 m/z

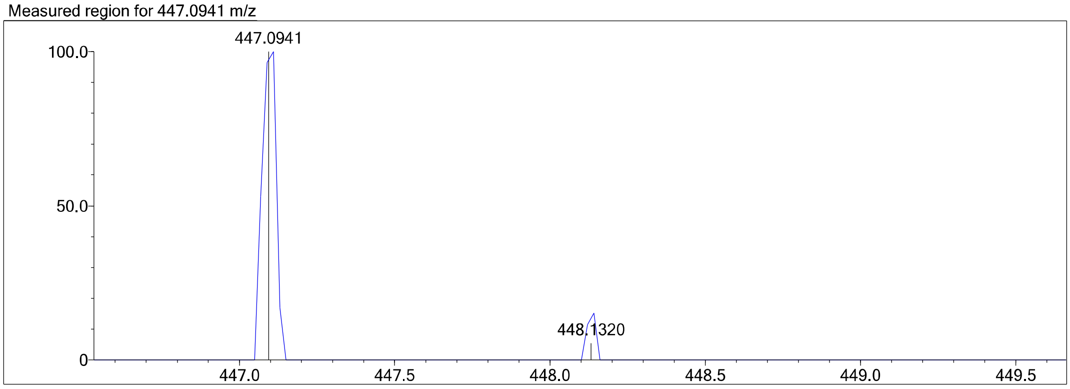

C21 H20 O11 [M-H]- : Predicted region for 447.0933 m/z

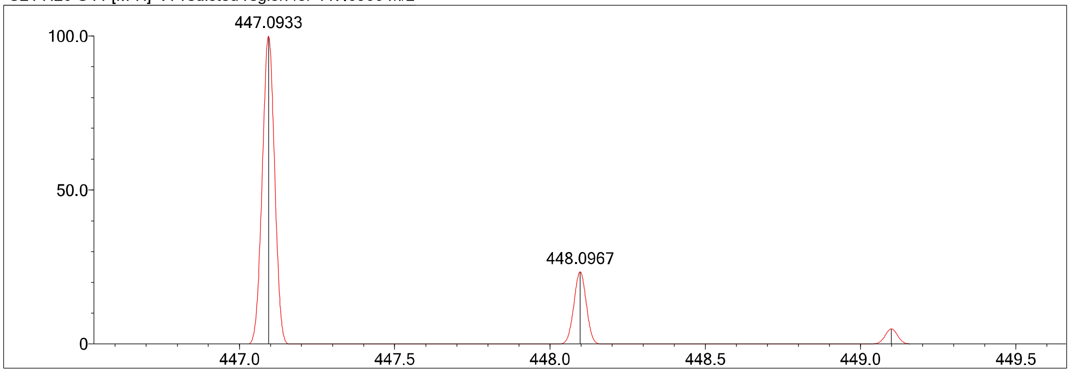

Rank	Score	Formula (M)	Ion	Meas. m/z	Pred. m/z	Df. (mDa)	Df. (ppm)	Iso	DBE
1	0.00	C21 H20 O11	[M-H]-	447.0941	447.0933	0.8	1.79	0.00	12.0

Figure S9. Cynaroside ESI (−) HRMS report

Data File: C:\LabSolutions\Data\Analiz\BETÜL AYDIN\NS-33-I_12.lcd

Elmt	Val.	Min	Max	Elmt	Val.	Min	Max	Elmt	Val.	Min	Max	Elmt	Val.	Min	Max	Use Adduct
H	1	10	36	O	2	0	6	S	2	0	0	Ru	2	0	0	H
C	4	10	28	F	1	0	0	Cl	1	0	0	Pd	2	0	0	
N	3	0	0	P	3	0	0	Br	1	0	0	I	3	0	0	

Error Margin (ppm):	5	DBE Range:	0.0 - 25.0	Electron Ions:	both
HC Ratio:	unlimited	Apply N Rule:	yes	Use MSn Info:	yes
Max Isotopes:	5	Isotope RI (%):	1.00	Isotope Res:	9000
MSn Iso RI (%):	10.00	MSn Logic Mode:	AND	Max Results:	50

Event#: 1 MS(E+) Ret. Time : 1.187 -> 1.627 Scan# : 179 -> 245

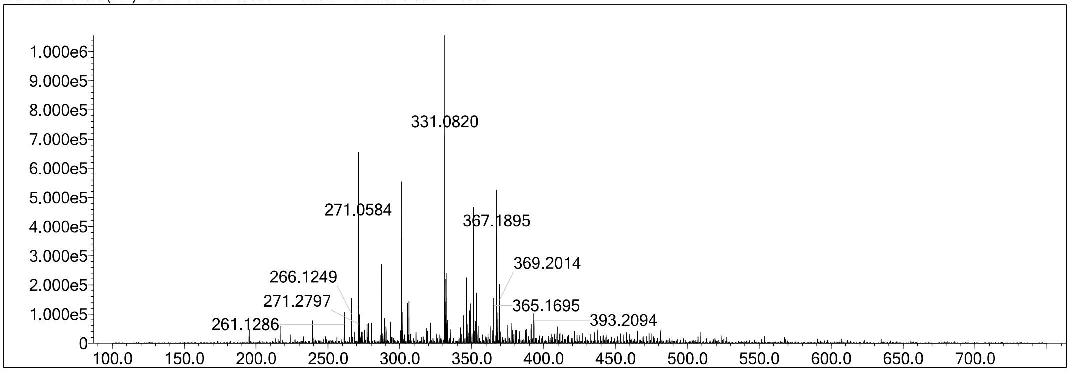

Measured region for 287.0549 m/z

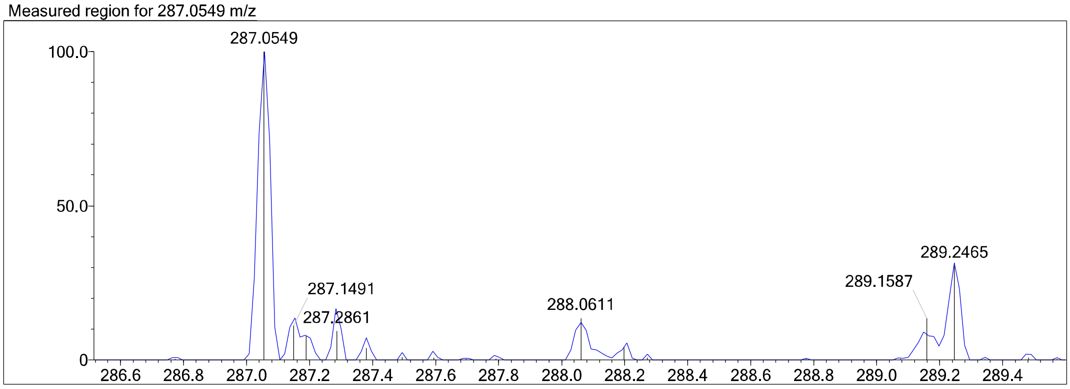

C15 H10 O6 [M+H]+ : Predicted region for 287.0550 m/z

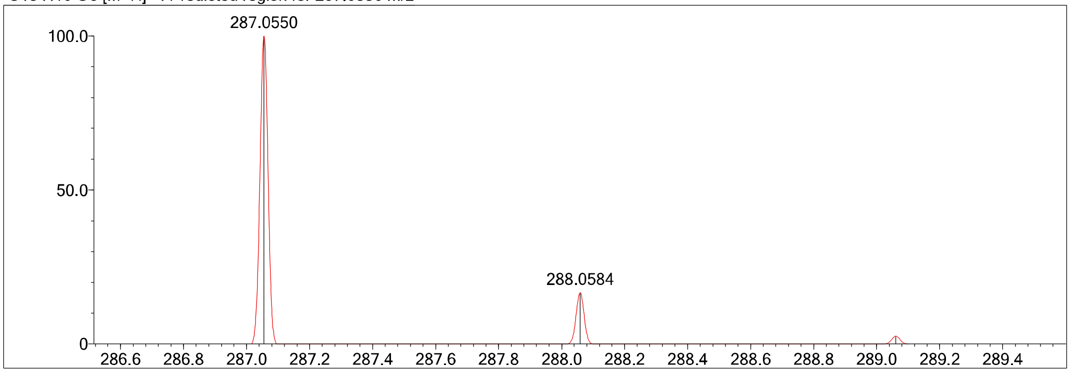

Rank	Score	Formula (M)	Ion	Meas. m/z	Pred. m/z	Df. (mDa)	Df. (ppm)	Iso	DBE
1	45.51	C15 H10 O6	[M+H]+	287.0549	287.0550	-0.1	-0.35	45.51	11.0

Figure S10. Luteolin ESI (+) HRMS report

Data File: C:\LabSolutions\Data\Analiz\BETÜL AYDIN\NS-33-I_12.lcd

Elmt	Val.	Min	Max	Elmt	Val.	Min	Max	Elmt	Val.	Min	Max	Elmt	Val.	Min	Max	Use Adduct
H	1	10	36	O	2	0	6	S	2	0	0	Ru	2	0	0	H
C	4	10	28	F	1	0	0	Cl	1	0	0	Pd	2	0	0	
N	3	0	0	P	3	0	0	Br	1	0	0	I	3	0	0	

Error Margin (ppm):	5	DBE Range:	0.0 - 25.0	Electron Ions:	both
HC Ratio:	unlimited	Apply N Rule:	yes	Use MSn Info:	yes
Max Isotopes:	5	Isotope RI (%):	1.00	Isotope Res:	9000
MSn Iso RI (%):	10.00	MSn Logic Mode:	AND	Max Results:	50

Event#: 2 MS(E-) Ret. Time : 1.307 -> 1.307 Scan# : 198 -> 198

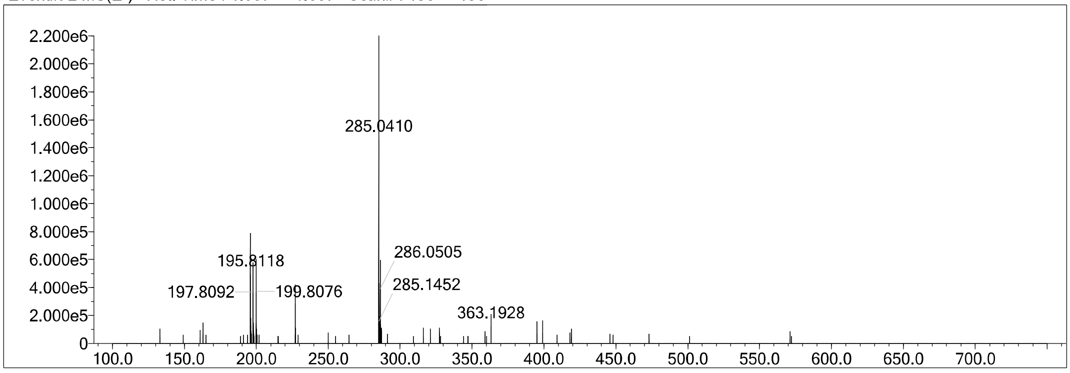

Measured region for 285.0410 m/z

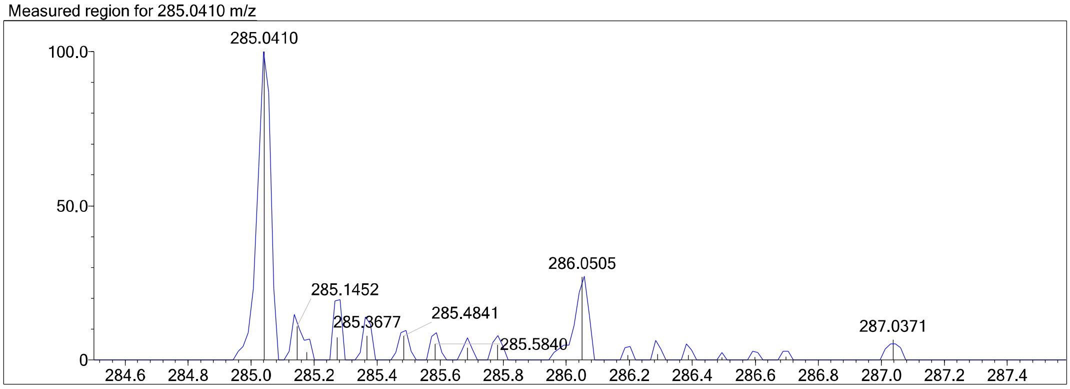

C15 H10 O6 [M-H]- : Predicted region for 285.0405 m/z

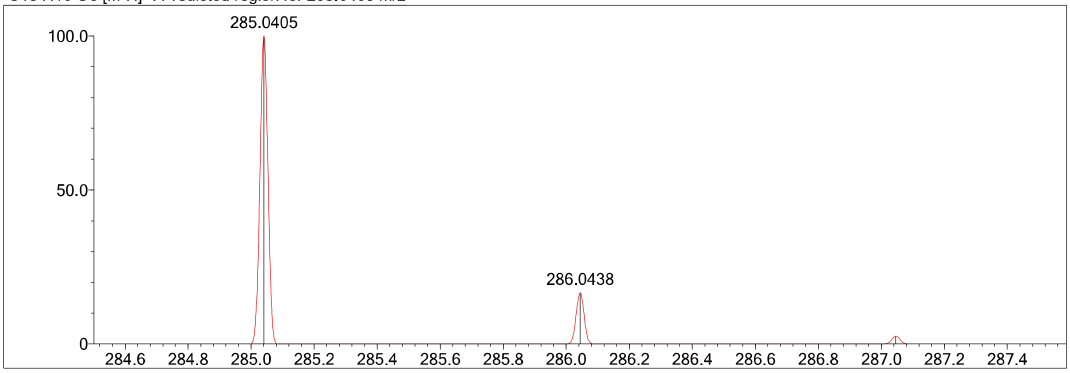

Rank	Score	Formula (M)	Ion	Meas. m/z	Pred. m/z	Df. (mDa)	Df. (ppm)	Iso	DBE
1	51.83	C15 H10 O6	[M-H]-	285.0410	285.0405	0.5	1.75	52.82	11.0

Figure S11. Luteolin ESI (−) HRMS report

Data File: C:\LabSolutions\Data\Analiz\BETÜL AYDIN\NS-35-E_17.lcd

Elmt	Val.	Min	Max	Elmt	Val.	Min	Max	Elmt	Val.	Min	Max	Elmt	Val.	Min	Max	Use Adduct
H	1	10	36	O	2	0	15	S	2	0	0	Ru	2	0	0	H
C	4	10	28	F	1	0	0	Cl	1	0	0	Pd	2	0	0	
N	3	0	0	P	3	0	0	Br	1	0	0	I	3	0	0	

Error Margin (ppm):	5	DBE Range:	0.0 - 25.0	Electron Ions:	both
HC Ratio:	unlimited	Apply N Rule:	yes	Use MSn Info:	yes
Max Isotopes:	5	Isotope RI (%):	1.00	Isotope Res:	9000
MSn Iso RI (%):	10.00	MSn Logic Mode:	AND	Max Results:	50

Event#: 1 MS(E+) Ret. Time : 0.933 -> 1.187 Scan# : 141 -> 179

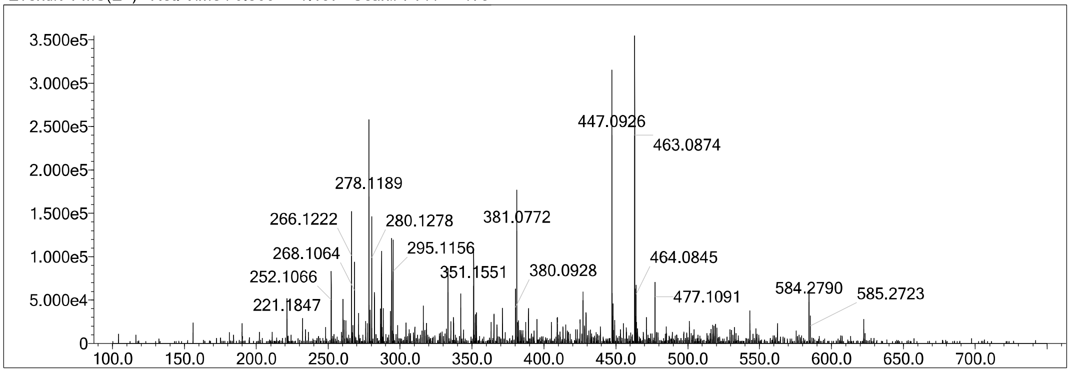

Measured region for 463.0874 m/z

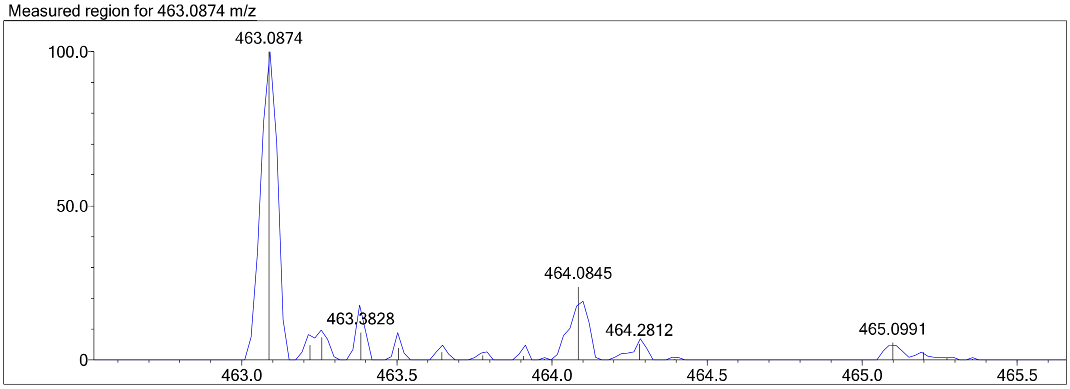

C21 H18 O12 [M+H]+ : Predicted region for 463.0871 m/z

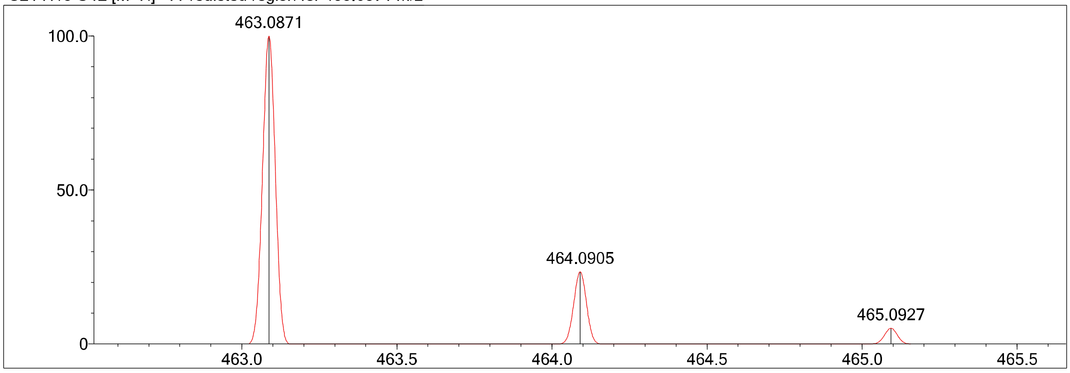

Rank	Score	Formula (M)	Ion	Meas. m/z	Pred. m/z	Df. (mDa)	Df. (ppm)	Iso	DBE
1	74.56	C21 H18 O12	[M+H]+	463.0874	463.0871	0.3	0.65	74.56	13.0

Figure S12. Luteolin-O- glucuronide ESI (+) HRMS report

Data File: C:\LabSolutions\Data\Analiz\BETÜL AYDIN\NS-31-I_10.lcd

Elmt	Val.	Min	Max	Elmt	Val.	Min	Max	Elmt	Val.	Min	Max	Elmt	Val.	Min	Max	Use Adduct
H	1	10	36	O	2	0	15	S	2	0	0	Ru	2	0	0	H
C	4	10	28	F	1	0	0	Cl	1	0	0	Pd	2	0	0	
N	3	0	0	P	3	0	0	Br	1	0	0	I	3	0	0	

Error Margin (ppm):	5	DBE Range:	0.0 - 25.0	Electron Ions:	both
HC Ratio:	unlimited	Apply N Rule:	yes	Use MSn Info:	yes
Max Isotopes:	5	Isotope RI (%):	1.00	Isotope Res:	9000
MSn Iso RI (%):	10.00	MSn Logic Mode:	AND	Max Results:	50

Event#: 1 MS(E+) Ret. Time : 2.293 Scan# : 345

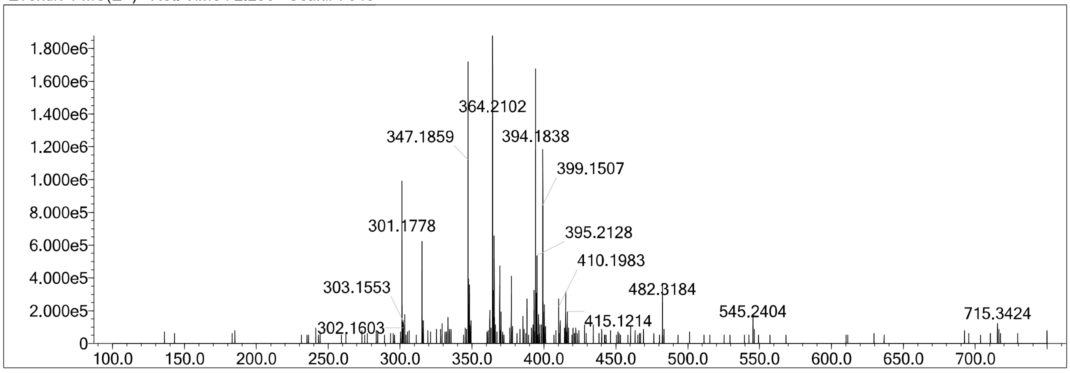

Measured region for 347.1859 m/z

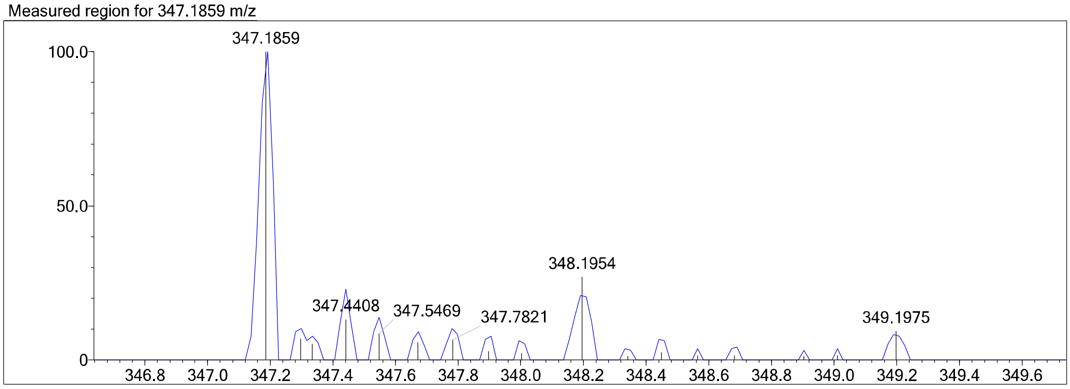

C20 H26 O5 [M+H]+ : Predicted region for 347.1853 m/z

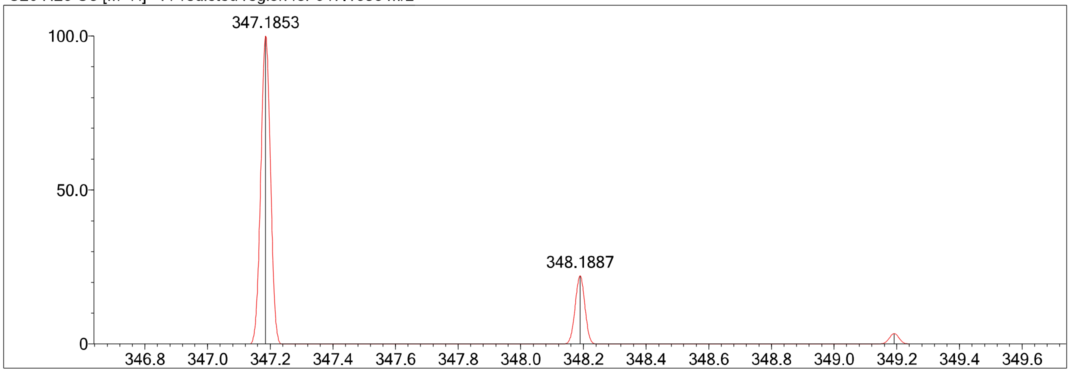

Rank	Score	Formula (M)	Ion	Meas. m/z	Pred. m/z	Df. (mDa)	Df. (ppm)	Iso	DBE
1	58.93	C20 H26 O5	[M+H]+	347.1859	347.1853	0.6	1.73	60.02	8.0

Figure S13. Rosmanol ESI (+) HRMS report

Data File: C:\LabSolutions\Data\Analiz\BETÜL AYDIN\NS-31-I_10.lcd

Elmt	Val.	Min	Max	Elmt	Val.	Min	Max	Elmt	Val.	Min	Max	Elmt	Val.	Min	Max	Use Adduct
H	1	10	36	O	2	0	15	S	2	0	0	Ru	2	0	0	H
C	4	10	28	F	1	0	0	Cl	1	0	0	Pd	2	0	0	
N	3	0	0	P	3	0	0	Br	1	0	0	I	3	0	0	

Error Margin (ppm):	5	DBE Range:	0.0 - 25.0	Electron Ions:	both
HC Ratio:	unlimited	Apply N Rule:	yes	Use MSn Info:	yes
Max Isotopes:	5	Isotope RI (%):	1.00	Isotope Res:	9000
MSn Iso RI (%):	10.00	MSn Logic Mode:	AND	Max Results:	50

Event#: 2 MS(E-) Ret. Time : 2.133 -> 2.373 Scan# : 322 -> 358

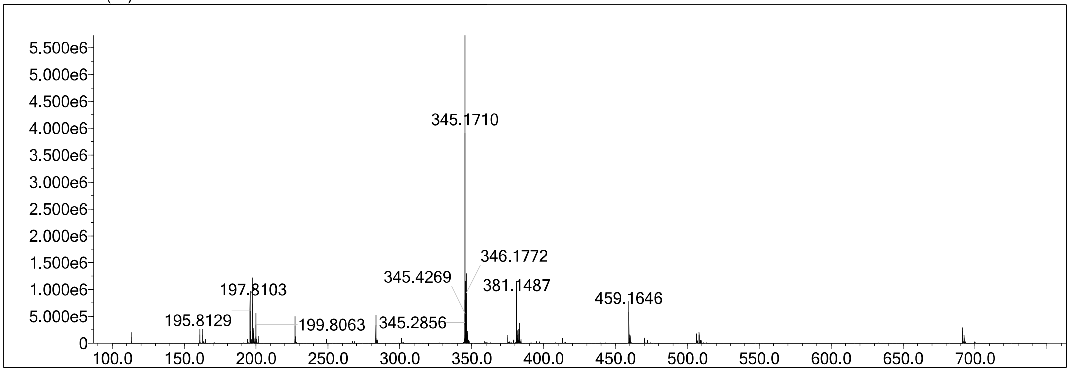

Measured region for 345.1710 m/z

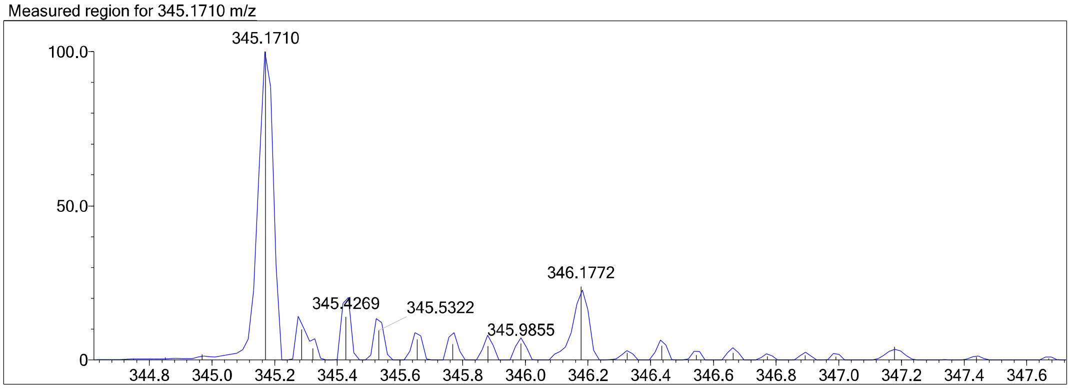

C20 H26 O5 [M-H]- : Predicted region for 345.1707 m/z

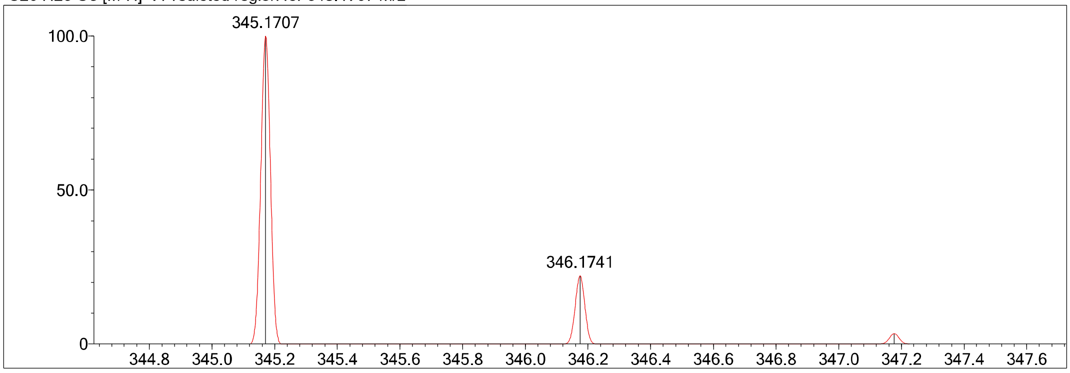

Rank	Score	Formula (M)	Ion	Meas. m/z	Pred. m/z	Df. (mDa)	Df. (ppm)	Iso	DBE
1	86.71	C20 H26 O5	[M-H]-	345.1710	345.1707	0.3	0.87	86.71	8.0

Figure S14. Rosmanol ESI (−) HRMS report

Data File: C:\LabSolutions\Data\Analiz\BETÜL AYDIN\SG-E_21.lcd

Elmt	Val.	Min	Max	Elmt	Val.	Min	Max	Elmt	Val.	Min	Max	Elmt	Val.	Min	Max	Use Adduct
H	1	10	36	O	2	0	15	S	2	0	0	Ru	2	0	0	H
C	4	10	28	F	1	0	0	Cl	1	0	0	Pd	2	0	0	
N	3	0	0	P	3	0	0	Br	1	0	0	I	3	0	0	

Error Margin (ppm): 5
HC Ratio: unlimited
Max Isotopes: 5
MSn Iso RI (%): 10.00

DBE Range: 0.0 - 25.0
Apply N Rule: yes
Isotope RI (%): 1.00
MSn Logic Mode: AND

Electron Ions: both
Use MSn Info: yes
Isotope Res: 9000
Max Results: 50

Event#: 2 MS(E-) Ret. Time : 1.187 Scan# : 180

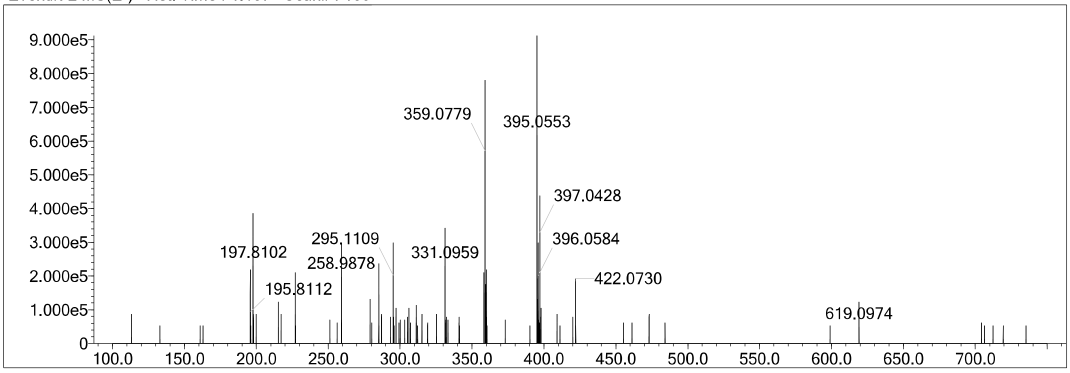

Measured region for 359.0779 m/z

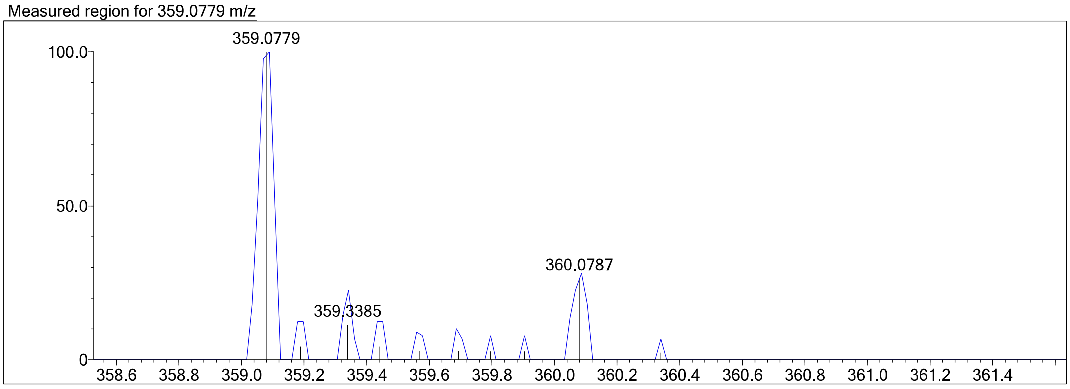

C18 H16 O8 [M-H]- : Predicted region for 359.0772 m/z

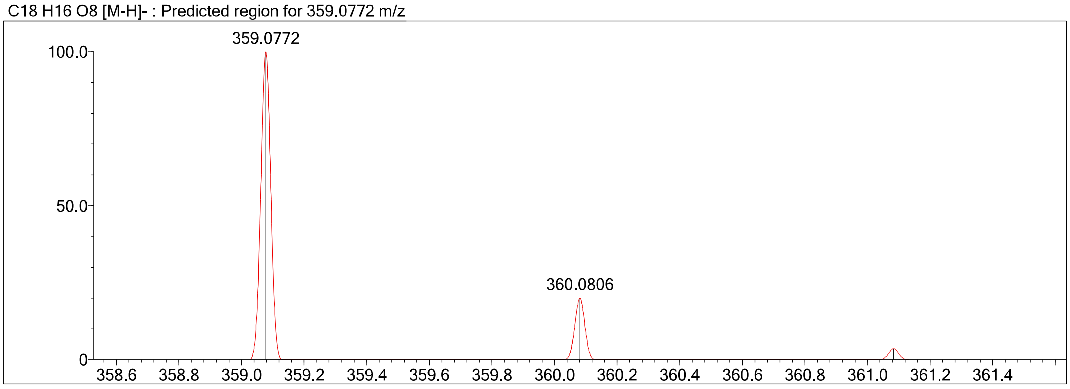

Rank	Score	Formula (M)	Ion	Meas. m/z	Pred. m/z	Df. (mDa)	Df. (ppm)	Iso	DBE
1	71.12	C18 H16 O8	[M-H]-	359.0779	359.0772	0.7	1.95	72.85	11.0

Figure S15. Rosmarinic acid ESI (−) HRMS report
